# p300 inhibition delays premature cellular senescence

**DOI:** 10.1038/s41514-025-00251-y

**Published:** 2025-07-10

**Authors:** Elisabetta Di Fede, Esi Taci, Silvia Castiglioni, Stefano Rebellato, Silvia Ancona, Paolo Grazioli, Chiara Parodi, Elisa Adele Colombo, Clara Bernardelli, Elena Lesma, Ian Daniel Krantz, Stefania Corti, Alberto Priori, Grazia Fazio, Cristina Gervasini, Valentina Massa, Antonella Lettieri

**Affiliations:** 1 Department of Health Sciences, Università degli Studi di Milano, Milan, Italy; 2 “Aldo Ravelli” Center for Neurotechnology and Experimental Brain Therapeutics, Università degli Studi di Milano, Milan, Italy; 3 Tettamanti Center, Fondazione IRCCS, San Gerardo dei Tintori, Monza, Italy; 4 School of Medicine and Surgery, University of Milano-Bicocca, Monza, Italy; 5 Division of Medical Genetics, Cohen Children’s Medical Center, Northwell Health, New York, NY USA; 6 Department of Pediatrics, Zucker School of Medicine, Hofstra University, New York, NY USA; 7 Dino Ferrari Centre, Department of Pathophysiology and Transplantation (DEPT), Neuroscience Section, University of Milan, Milan, Italy; 8 IRCCS Foundation Ca’ Granda Ospedale Maggiore Policlinico, Milan, Italy

**Keywords:** Diseases, Pathogenesis, Cell biology, Senescence

## Abstract

Cellular senescence represents a permanent state of cell cycle arrest, also observed in neurodegenerative disorders. As p300 has been identified as an epigenetic driver of replicative senescence, we aimed to investigate whether in vitro p300 inhibition could rescue the stress-induced premature senescence (SIPS) phenotype. We exploited 2D and 3D (brain organoids) in vitro models of SIPS using two different stressor agents. In addition, we combined the treatment with a p300 inhibitor and validated p300 role in SIPS by analyzing different senescence markers and the transcriptome in our models. Interestingly, p300 inhibition can counteract the DNA damage and SIPS phenotype, detecting a dysregulation of gene expression and protein translation associated with the senescence program. These findings highlight both the molecular mechanisms underlying senescence and p300 as a possible pharmacological target. Thus, targeting p300 and, by extension, senescent cells could represent a promising therapeutic strategy for age-related diseases such as neurodegenerative disorders.

## Introduction

Cellular senescence represents a state of cell cycle arrest occurring when cells are at the end of their replicative lifespan (i.e., replicative senescence), or are induced into this permanent state by several stressors (i.e., stress-induced premature senescence, SIPS), including reactive oxygen species (ROS, e.g., hydrogen peroxide), genotoxic agents (e.g., cisplatin, doxorubicin, taxol, vincristine) or oncogene activation^1–4^. Senescence has been recognized in both physiological processes such as tissue repair, tumor suppression and embryonic development, and pathological ones such as tumorigenesis and age-related pathologies^5–7^. In the last decade, it has become more evident that aging is characterized by the accumulation and persistence of senescent cells, which in physiological conditions are usually induced in a transient and regulated manner^8^. On the other hand, the detrimental presence of senescent cells is responsible for the induction of an inflammatory state, also due to chronic SASP (senescence-associated secretory phenotype) production, which can lead to tissue dysfunction and contribute to age-related disorders^9,10^. In addition, senescent-like phenotypes have been observed not only in proliferating cells but also in post-mitotic cell types, including neurons, contributing to pathological features underlying neurodegenerative disorders^11,12^. Indeed, growing evidence suggests that senescent features are present in brain tissues and in vitro or in vivo models of Parkinson’s disease (PD)^13–16^, amyotrophic lateral sclerosis (ALS)^17^, Alzheimer’s disease (AD)^18–20^ and other neurodegenerative disorders (e.g., progressive supranuclear palsy and traumatic brain injury)^18^.

Senescent cells undergo phenotypic and metabolic changes such as a typical flat morphology, resistance to apoptosis, chromatin remodeling (senescence-associated heterochromatic foci or SAHF), secretion of inflammatory cytokines known as SASP, higher expression of proteins belonging to tumor suppressor network (p21/p53 and p16/RB) and senescence-associated β-galactosidase (SA-β-gal) activation^7,21^. Several senescence biomarkers have been identified, highlighting the number of actors playing in molecular pathways underlying this process. However, to date, the molecules at the beginning of the cascade remain poorly understood^22^. As an alteration of the epigenome is known to characterize cellular senescence, recent findings on the acetyltransferase p300, identified as the epigenetic driver of replicative senescence, could represent a turning point in the field^23,24^. The authors characterized a molecular mechanism underlying senescence, showing that p300 induces super-enhancers responsible for senescent-specific gene expression program activation^23^. Thus, targeting p300 and, by extension, senescent cells could represent a promising strategy for age-related diseases.

In this work, we utilized both 2D and 3D in vitro models to determine whether p300 genetic haploinsufficiency and chemical p300 inhibition could rescue DNA damage and SIPS-related phenotypes. We used SIPS agents and validated the role of p300 in lymphoblastoid cell lines (LCLs) derived from patients affected by a rare genetic disorder with pathogenetic variants in *EP300*, the gene coding for p300, and healthy donors (HD). We then focused on p300 chemical inhibition on induced senescent-like phenotypes in different 2D models such as HD LCLs, primary lung fibroblasts and iNeurons differentiated from human induced pluripotent stem cells (hiPSC) obtained from a healthy donor. We also investigated the effect of p300 inhibition in more complex 3D neuronal cultures such as brain organoids (BOs) generated from control hiPSC, which met the needs for recapitulating in vitro the human complexity and physiology relevant for disease modeling and drug discovery^25^.

## Results

### LCLs with germline pathogenic variants in *EP300* are less responsive to DNA damage induced by SIPS agents

**Fig. 1:**
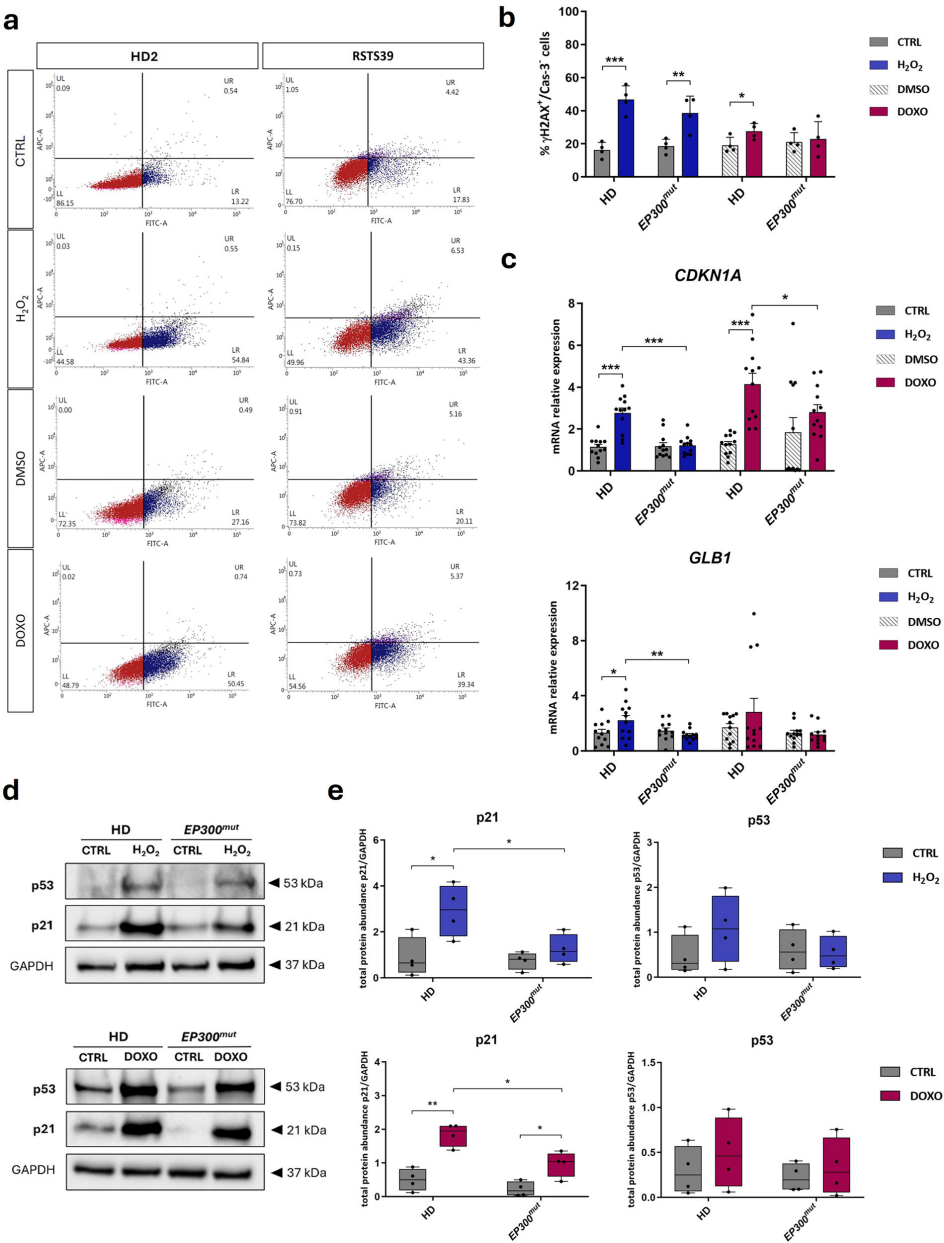
*EP300*^*mut*^ LCLs are less sensitive to DNA damage induced by SIPS agents. **a** Representative flow cytometry panel for γH2AX (FITC-A) and cleaved Caspase 3 (APC-A) evaluation of LCLs derived from one individual (RSTS39) of the cohort with germline pathogenic variants in *EP300* (*EP300*^*mut*^) and from one individual (HD2) of the control cohort (HD) both untreated (CTRL), treated with hydrogen peroxide (H_2_O_2_), doxorubicin (DOXO) or its vehicle (DMSO); for each cell line there are four quadrants showing percentage of cells negative for both APC and FITC signals (lower left, quadrant LL), positive for APC and negative for FITC signal (upper left quadrant, UL), positive for both (upper right quadrant, UR) or negative for APC and positive for FITC signal (lower right quadrant, LR). **b** Flow cytometry analysis showing percentage of cells positive to γH2AX and not to cleaved Caspase 3 (% γH2AX^+^/Cas-3^-^ cells, on Y axis) on *EP300*^*mut*^ and HD lines untreated (CTRL, dark gray), treated with hydrogen peroxide (H_2_O_2_, blue), DMSO (gray stripes) or doxorubicin (DOXO, plum); each dot represents the mean of triplicates of *n* = 4 cell lines and values are expressed as means ± SD. **c** mRNA relative expression of senescence markers *CDKN1A* and *GLB1* in LCLs bearing a pathogenic variant in *EP300* (*EP300*^*mut*^) and controls (HD) LCLs exposed to SIPS agents such as hydrogen peroxide (H_2_O_2_ in blue) or doxorubicin (DOXO in plum), expressed as fold change calculated on their respective controls (untreated in dark gray and treated with DMSO in gray stripes); dots express triplicates of the four lines (*n* = 12) and values are expressed as means ± SEM. Representative western blot showing total protein abundance of senescent markers p21 and p53 normalized on GAPDH (**d**) and replicates analysis (**e**) on LCLs with pathogenic variant in *EP300* (*EP300*^*mut*^) and controls (HD) LCLs not treated (CTRL, dark gray) or exposed to hydrogen peroxide (H_2_O_2_ in blue) or doxorubicin (DOXO in plum); dots represent *n* = 4 replicates and values are expressed as means ± SEM. Statistical analysis was performed using two-tailed Student *t*-test (*p < 0.05; **p < 0.01; ***p < 0.001).

To confirm the role of p300 in cellular senescence, we investigated the response to DNA damage induced by two different SIPS agents in an in vitro model of LCLs derived from patients with Rubinstein-Taybi syndrome type 2 (RSTS2, OMIM #613684) carrying germline mutations in *EP300* (*EP300*^*mut*^) (Table 1, see Methods), which result in haploinsufficiency or defective p300 function^26^ (Fig. 1). We treated four *EP300*^*mut*^ LCLs and four LCLs derived from healthy donors (HD) with two SIPS agents: the ROS hydrogen peroxide (H_2_O_2_) and the intercalating and chemotherapeutic agent doxorubicin (DOXO)^27–30^ (Table 2, see Methods).

We analyzed the DNA damage marker γH2AX by flow cytometry and the apoptosis marker cleaved Cas-3 in all the treated and untreated LCLs, focusing on the senescent-like phenotype identified by positivity to γH2AX and not to cleaved Cas-3 (γH2AX^+^/Cas-3^-^ cells) (Fig. 1a, b and Supplementary Fig. 1). We observed significant SIPS induction for H_2_O_2_ in both HD (*p* < 0.001) and *EP300*^*mut*^ (*p* < 0.01) LCLs compared to the untreated one (CTRL) or vehicle (DMSO) for DOXO treatment in HD LCLs (*p* < 0.05) (Fig. 1b). Interestingly, the percentage of γH2AX^+^/Cas-3^−^ cells in DOXO treatment in *EP300*^*mut*^ LCLs was comparable to the DMSO group, and with H_2_O_2_-treatment *EP300*^*mut*^ LCLs showed less DNA damage than that in HD LCLs (Fig. 1b).

Furthermore, using RT-qPCR, we observed higher expression of senescent markers such as *CDKN1A*, coding for p21, and *GLB1*, coding for galactosidase beta 1, in HD LCLs treated with H_2_O_2_ (*p* < 0.001 for *CDKN1A* and *p* < 0.01 for *GLB1*) or DOXO (*p* < 0.001 for *CDKN1A*) compared to their respective control group (Fig. 1c). Additionally, *EP300*^*mut*^ LCLs show no significant increase in *CDKN1A* or *GLB1* expression when treated with H_2_O_2_ or DOXO (Fig. 1c). Notably, the expression of *CDKN1A* and *GLB1* in HD LCLs was significantly higher than in *EP300*^*mut*^ LCLs when both groups were treated with SIPS agents (*p* < 0.001 for *CDKN1A* and *p* < 0.01 for *GLB1* in H_2_O_2_-treated groups; *p* < 0.05 for *CDKN1A* in DOXO-treated groups) (Fig. 1c).

Subsequently, we investigated senescence markers at protein levels by performing western blot on *EP300*^*mut*^ and HD LCL lysates (Fig. 1d and Supplementary Fig. 2a). We confirmed a significant increase of p21 in HD LCLs when treated with H_2_O_2_ (*p* < 0.05) and in both *EP300*^*mut*^ and HD LCLs upon DOXO treatment (*p* < 0.05 and *p* < 0.01 respectively) compared to untreated samples. In addition, an increased trend in p53 expression was observed in both HD LCLs treated samples compared to their controls (Fig. 1e). However, the increase of senescence markers expression appeared to be lower in *EP300*^*mut*^-treated than in HD-treated LCLs, significantly for p21 abundance (*p* < 0.05 for both SIPS agents treatment) (Fig. 1e). All these data suggest that *EP300*^*mut*^ LCLs are less sensitive to DNA damage induced by SIPS agents, confirming the possible pivotal role of p300 in the senescence cascade.

### p300 inhibition by CCS1477 rescues the induced DNA damage by SIPS agents in HD LCLs

**Fig. 2:**
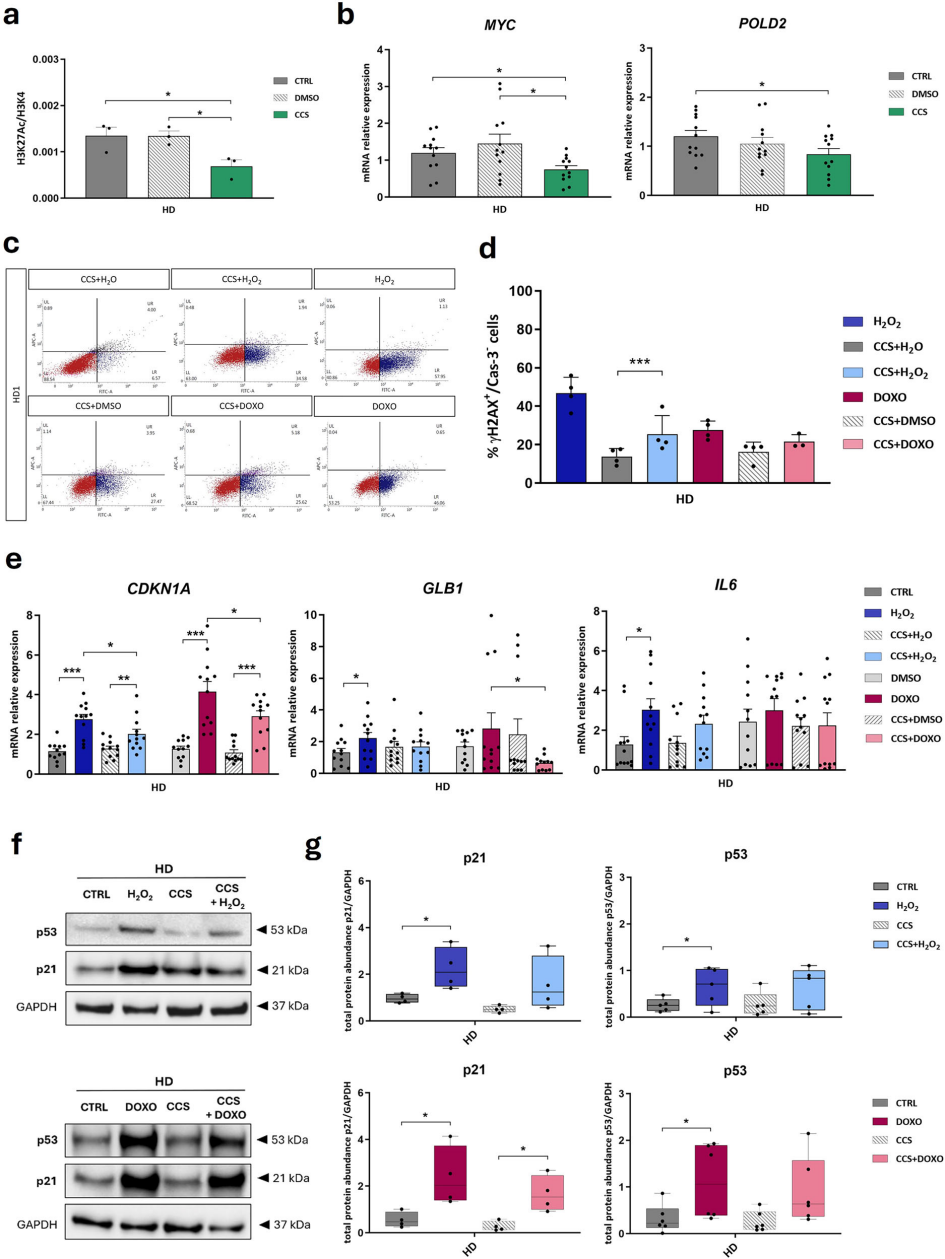
p300 inhibition by CCS1477 protects HD LCLs from DNA damage induced by SIPS agents. **a** Acetylation of lysine 27 of histone 3 (H3K27Ac) normalized on unmodified lysine 4 of histone 3 (H3K4) (H3K27Ac/H3K4, on Y-axis) in LCLs derived from three healthy donors (HD) not treated (CTRL, dark gray bar), treated with vehicle (DMSO, gray striped bar) or with CCS1477 (CCS, green bar), assessed by AlphaLISA assay; dots show *n* = 3 biological triplicates and values are expressed as means ± SEM. **b** mRNA relative expression of known p300 transcriptional targets *MYC* and *POLD2* in all healthy donors LCLs (HD) untreated (CTRL, dark gray bar), treated with vehicle (DMSO, gray striped bar) or with CCS1477 (CCS, green bar); dots express triplicates of the four lines (*n* = 12) and values are expressed as means ± SEM. **c** Representative flow cytometry panel for γH2AX (FITC-A) and cleaved Caspase 3 (APC-A) evaluation of one control LCL (HD1) treated with hydrogen peroxide alone (H_2_O_2_) or combined with CCS1477 (CCS + H_2_O_2_), treated with its vehicle (CCS + H_2_O), with doxorubicin alone (DOXO) or combined with CCS1477 (CCS + DOXO) or treated with its vehicle (CCS + DMSO); for each cell line there are four quadrants showing percentage of cells negative for both APC and FITC signals (lower left, quadrant LL), positive for APC and negative for FITC signal (upper left quadrant, UL), positive for both (upper right quadrant, UR) or negative for APC and positive for FITC signal (lower right quadrant, LR). **d** Flow cytometry analysis showing percentage of cells positive for γH2AX and not to cleaved Caspase 3 (% γH2AX^+^/Cas-3^-^ cells, on Y axis) on controls lines (HD) treated with hydrogen peroxide alone (H_2_O_2_, blue bar) or combined with CCS1477 (CCS + H_2_O_2_, light blue bar), treated with its vehicle (CCS + H_2_O, gray bar), with doxorubicin alone (DOXO, plum bar) or combined with CCS1477 (CCS + DOXO, pink bar) or treated with its vehicle (CCS + DMSO, gray striped bar); each dot represents the mean of triplicates of *n* = 4 cell lines and values are expressed as means ± SD. **e** mRNA relative expression of senescence markers *CDKN1A*, *IL6* and *GLB1* in controls LCLs (HD) exposed to SIPS agents such as hydrogen peroxide (H_2_O_2_ in blue) or doxorubicin (DOXO in plum) alone or combined with CCS1477 (CCS + H_2_O_2_, light blue bar; CCS + DOXO, pink bar), expressed as fold change calculated on their respective controls (untreated, treated with DMSO, CCS + H_2_O or CCS + DMSO which values are represented by the dotted line); dots express triplicates of the four lines (*n* = 12) and values are expressed as means ± SEM. Representative western blot showing total protein abundance of senescent markers p21 and p53 normalized on GAPDH (**f**) and replicates analysis (**g**) on controls LCLs (HD) untreated (CTRL, dark gray bar) or exposed to hydrogen peroxide (H_2_O_2_, blue bar), doxorubicin (DOXO, plum bar), CCS1477 (CCS, gray striped bar) or SIPS agents in combination with CCS (CCS + H_2_O_2_, light blue bar; CCS + DOXO, pink bar); dots represent *n* = 4 replicates and values are expressed as means ± SEM. Statistical analysis was performed using two-tailed Student *t*-test (*p < 0.05; **p < 0.01; ***p < 0.001).

We then decided to mimic p300 deficiency chemically by bromodomain inhibitor of p300 known as CCS1477 (CCS) on different models upon SIPS induction, starting from HD LCLs (Fig. 2 and Table 2, see Methods).

Firstly, we verified the effect of the inhibitor on p300, assessing both the acetylation levels and the gene expression of p300 transcriptional targets in HD LCLs treated with CCS, with its vehicle (DMSO), or not treated (CTRL) (Fig. 2a, b). In detail, levels of acetylated lysine 27 of histone H3 (H3K27ac) assessed by AlphaLISA assay were significantly reduced in HD LCLs where p300 was inhibited (CCS) compared to their respective controls (*p* < 0.05) (Fig. 2a). In addition, the expression of p300 transcriptional targets such as *MYC* and *POLD2* decreased when cells were treated with CCS, this reached significance for HD LCLs compared to its control (*p* < 0.05 for both *MYC* and *POLD2*) (Fig. 2b). The same effect was observed in IMR90 cells, a well-known in vitro model of senescence (Supplementary Fig. 3). Hence, we decided to combine treatments with CCS and SIPS agents (CCS + H_2_O_2_ or CCS + DOXO) to test whether p300 inhibition by CCS1477 could ameliorate DNA damage and, eventually, the senescent-like phenotype in all experimental models with SIPS induction.

Using flow cytometry, we evaluated the percentage of γH2AX^+^/Cas-3^−^ cells confirming that CCS treatment did not affect cell viability (Supplementary Fig. 4), as shown by CCS treatment in combination with SIPS agents’ vehicles (CCS + H_2_O or CCS + DMSO) compared to its combination with SIPS agents (CCS + H_2_O_2_ or CCS + DOXO) (*p* < 0.001 for H_2_O_2_-treatment). Importantly, we observed a trend in which a combination of CCS and SIPS agents seemed to ameliorate DNA damage compared to the one induced by H_2_O_2_ or DOXO alone (Fig. 2c, d).

When we analyzed gene expression of SIPS markers such as *CDKN1A*, *GLB1* and *IL6* by RT-qPCR, the trend observed in flow cytometry for γH2AX^+^/Cas-3^-^ cells was confirmed (Fig. 2e). SIPS agents caused an increased *CDKN1A*, *GLB1* and *IL6* expression significant for *CDKN1A* (*p* < 0.001 for H_2_O_2_ and DOXO, *p* < 0.01 for CCS + H_2_O_2_ and *p* < 0.001 for CCS + DOXO compared to their respective vehicles) and for *GLB1* and *IL6* in only H_2_O_2_ treatment (*p* < 0.05), confirming the DNA damage induced by SIPS detected by flow cytometry. In addition, significant differences in SIPS markers expression when p300 is inhibited were observed between HD LCLs treated with H_2_O_2_ and CCS + H_2_O_2_ (*p* < 0.05 for *CDKN1A*) and between HD LCLs treated with DOXO and CCS + DOXO (*p* < 0.05 for *CDKN1A* and *GLB1*) (Fig. 2e).

We then performed western blots on lysates of HD LCLs exposed to SIPS agents alone or combined with CCS to check the total protein abundance of senescence markers such as p21 and p53 (Fig. 2f and Supplementary Fig. 2b). We observed a significantly increased level of p21 and p53 in both H_2_O_2_- and DOXO-treated HD LCLs compared to controls (*p* < 0.05) and in combined treatments (CCS + H_2_O_2_ or CCS + DOXO) compared to CCS alone (significant only for p21 in CCS + DOXO samples), as expected (Fig. 2g). Interestingly, a slightly less abundance of p21 and p53 was shown in samples treated with SIPS in combination with CCS (CCS + H_2_O_2_ or CCS + DOXO) than in proteins obtained from LCLs exposed only to SIPS (H2O2 or DOXO) (Fig. 2g).

This in vitro model for assessing CCS11477 action in LCLs provides the first evidence for rescuing the DNA damage phenotype induced by SIPS agents.

### p300 inhibition by CCS1477 counteracts senescent-like phenotype in iNeurons

**Fig. 3:**
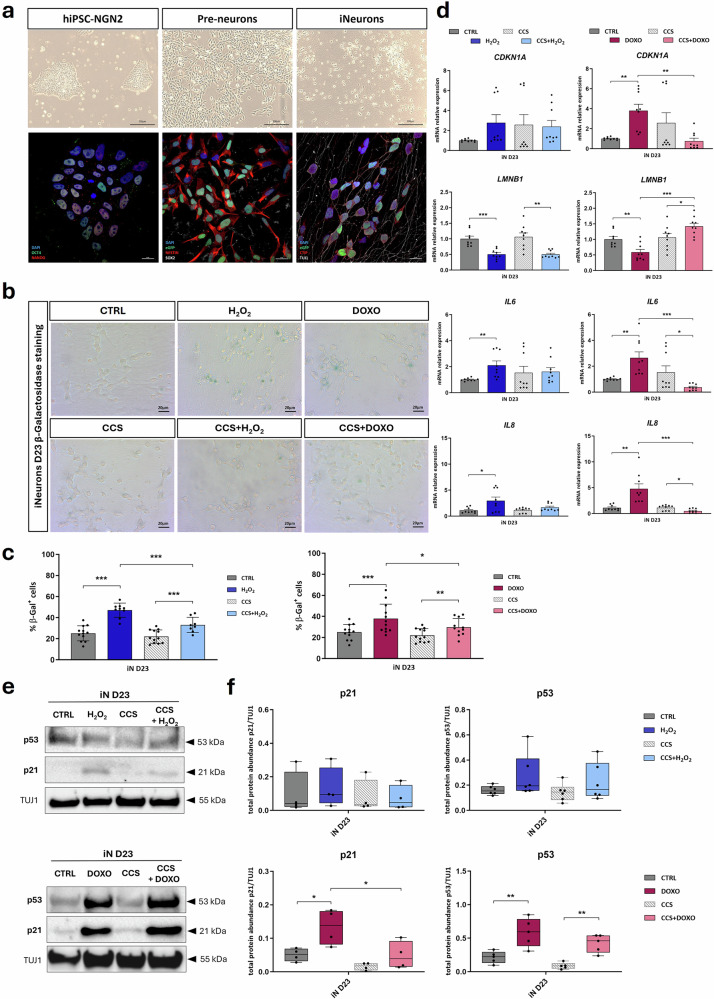
p300 inhibition by CCS1477 rescues the senescent-like phenotype induced by SIPS agents in iNeurons. **a** Characterization of the three main differentiation stages of iNeurons (hiPSC-NGN2, Pre-neurons and iNeurons) by brightfield acquisitions at 10x magnification (upper panel) and by immunofluorescence experiments acquired using a confocal microscope with 60x magnification (bottom panel): confocal images show hiPSCs tagged with OCT4 (green signal) and NANOG (red signal), Pre-neurons with NESTIN (red signal) and SOX2 (white signal), iNeurons with CTIP (red signal) and TUJ1 (white signal), all nuclei with DAPI (blue signal), while endogenous GFP due to inducible differentiation (eGFP, green signal) was detected in both Pre-Neurons and iNeurons. **b** Representative panels of SA-β-Gal staining on D23 iNeurons untreated (CTRL), treated with CCS1477 (CCS), with SIPS agents alone (H_2_O_2_ or DOXO) or in combination with CCS (CCS+H_2_O_2_or CCS + DOXO); brightfield images acquired at 20x magnification show cells positive for staining as blue-colored. **c** Analysis of iNeurons percentage positive to SA-β-Gal (% β-Gal+ cells, on Y-axis) untreated (CTRL, dark gray bar), treated with CCS (gray striped bar), with H_2_O_2_-treatments (H_2_O_2_, blue bar; CCS + H_2_O_2_, light blue bar) or DOXO-treatments (DOXO, plum bar; CCS + DOXO pink bar); dots show mean of blind fields counts of *n* = 3 biological replicates and values are expressed as means ± SD. **d** mRNA relative expression of senescence markers *CDKN1A*, *LMNB1*, *IL6* and *GLB1* in iNeurons exposed to SIPS agents alone (H_2_O_2_, blue bar or DOXO, plum bar) or combined with CCS1477 (CCS + H_2_O_2_, light blue bar; CCS + DOXO, pink bar), expressed as fold change calculated on their respective controls (untreated CTRL, dark gray bar and CCS, gray striped bar); dots express biological triplicates (*n* = 9) and values are expressed as means ± SEM. Representative western blots showing total protein abundance of senescent markers p21 and p53 normalized to TUJ1 (**e**) and replicates analysis (**f**) on iNeurons (iN D23) untreated (CTRL, dark gray bar) or exposed to H_2_O_2_ (blue bar), DOXO (plum bar), CCS (gray striped bar) or SIPS agents in combination with CCS (CCS + H_2_O_2_, light blue bar; CCS + DOXO, pink bar); dots represent at least *n* = 4 replicates and values are expressed as means ± SEM. Statistical analysis was performed using a two-tailed Student *t*-test (*p < 0.05; **p < 0.01; ***p < 0.001).

Considering the relevance of senescence in age-related disorders, especially neurodegenerative diseases, we wanted to test whether inhibition of p300 by CCS1477 could rescue the senescent-like phenotype in a neuronal context, exploiting iNeurons differentiated from our engineered hiPSC-NGN2 (Fig. 3). The construct contained in the hiPSC-NGN2 stable line allowed for doxycycline-inducible differentiation with cells expressing GFP in pre-neurons (corresponding to neural precursors) and iNeurons (corresponding to mature neurons) stages (Fig. 3a). After assessing neuronal markers during differentiation (NESTIN and SOX2 for neural precursors, CTIP and TUJ1 for cortical neurons, Fig. 3a and Supplementary Fig. 5a) and establishing their aging by expression of p300 transcriptional target (Supplementary Fig. 5b)^31^, in this 2D model at the D23 iNeurons stage, we induced senescence using SIPS agents (H_2_O_2_ or DOXO) and tested CCS effects (CCS, CCS + H_2_O_2_ or CCS + DOXO), as done for HD LCLs samples (Table 2, see Methods).

Firstly, we assessed the senescence-associated β-galactosidase (SA-β-Gal) by staining (Fig. 3b), observing a significant increase in the percentage of SA-β-Gal positive iNeurons when exposed to SIPS agents alone (*p* < 0.001 for H_2_O_2_ and DOXO), or in combination with CCS (*p* < 0.001 for CCS + H_2_O_2_ and *p* < 0.01 for CCS + DOXO) compared to their respective controls (untreated cells and treated with CCS only) (Fig. 3c). Interestingly, cells exposed to SIPS in combination with CCS showed a significant decrease in the percentage of β-galactosidase positive iNeurons compared to the ones treated with H_2_O_2_ or DOXO alone (*p* < 0.001 for H_2_O_2_-treated and *p* < 0.05 for DOXO-treated iNeurons) (Fig. 3c).

We subsequently investigated senescence markers at the transcriptional level performing RT-qPCR for *CDKN1A*, *LMNB1*, *IL6* and *IL8* (Fig. 3d), checking also the expression of p300 targets (Supplementary Fig. 6a). As expected, SIPS agents induced increased expression of *CDKN1A*, *IL6* and *IL8* in those iNeurons exposed to H_2_O_2_ (*p* < 0.01 for *IL6* and *p* < 0.05 for *IL8*) or DOXO (*p* < 0.01 for *CDKN1A*, *IL6* and *IL8*), together with a decrease of *LMNB1* mRNA relative expression (*p* < 0.001 for H_2_O_2_ and *p* < 0.01 for DOXO) compared to their untreated condition (Fig. 3d). Notably, a treatment combination of SIPS with p300 inhibitor led to a lower level of *CDKN1A*, *IL6* and *IL8* expression, and a higher one for *LMNB1*, all significant for the DOXO-treatments (*p* < 0.01 for *CDKN1A*, *p* < 0.001 for *LMNB1*, *IL6* and *IL8*), compared to SIPS agents-treatment alone (Fig. 3d).

We also determined p21 and p53 expression on lysates obtained from iNeurons by western blots (Fig. 3e and Supplementary Fig. 7a). As expected, H_2_O_2_- and DOXO-treatment in iNeurons caused an increase of senescence markers, more appreciable when cells were treated with DOXO alone compared to untreated samples (*p* < 0.05 for p21 expression and *p* < 0.01 for p53 expression) or in combination with CCS (*p* < 0.01 for CCS + DOXO compared to CCS-treated iNeurons for p53 expression) (Fig. 3f). A reduced abundance of senescence markers, also assessed by immunofluorescence performed for p21 (Supplementary Fig. 8), could be appreciated when iNeurons were treated in combination with CCS (CCS + H_2_O_2_ and CCS + DOXO) compared to SIPS agents alone, significant for DOXO-treated iNeurons (*p* < 0.05 for p21 expression) (Fig. 3f).

These data on a neuronal 2D in vitro model support the hypothesis that a senescent-like phenotype induced by SIPS agents could be counteracted through p300 inhibition by CCS1477 in iNeurons.

### p300 inhibition by CCS1477 counteracts senescent-like phenotype in Brain Organoids (BOs)

**Fig. 4:**
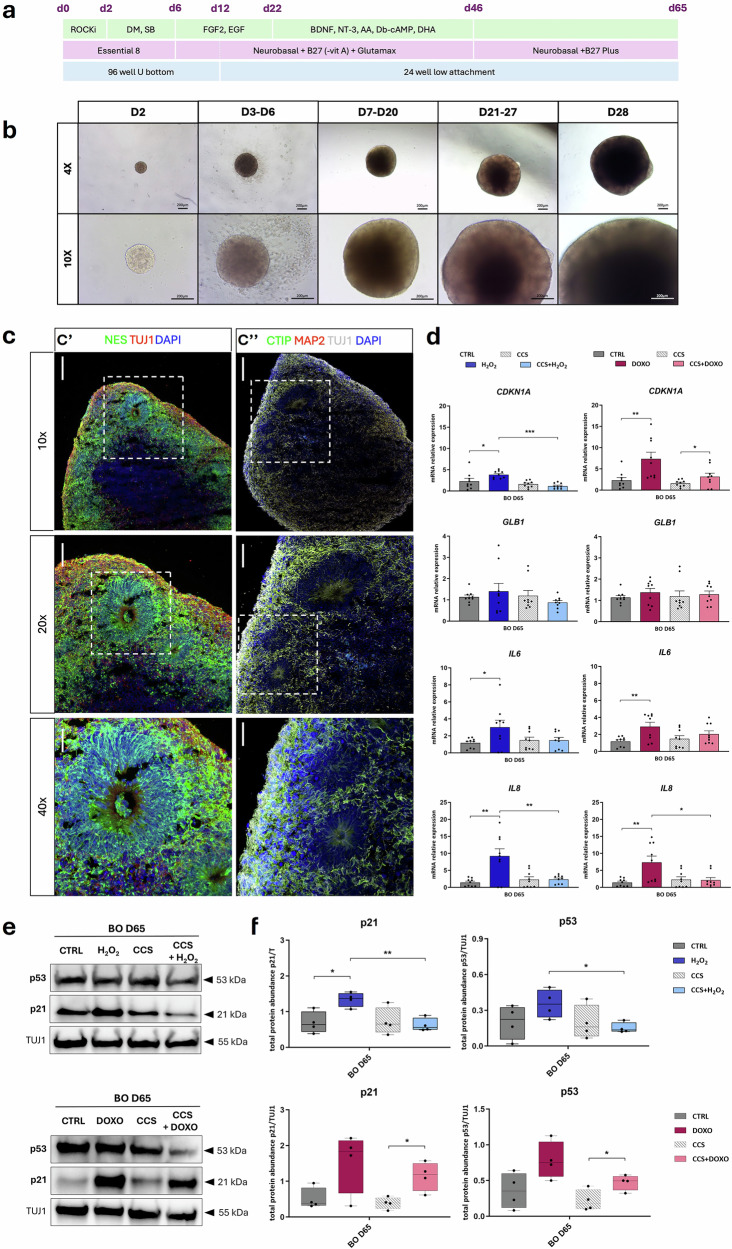
p300 inhibition by CCS1477 rescues the senescent-like phenotype induced by SIPS agents in Brain Organoids (BOs). **a** Scheme of organoid differentiation protocol starting from hiPSC to d65-organoid. **b** Brightfield images of brain organoids at different stages of differentiation at 4× and 10× magnification. **c** Characterization of brain organoids at d65 by immunofluorescence experiments; **c’**,**c”** 20 μm brain organoid sections immunolabelled with anti-NESTIN antibody (green) and anti-TUJ1 antibody (red) (**c’**), and anti-CITIP (green), anti-MAP2 (red) and anti-TUJ1 (white) (**c”**) at 10X, 20X and 40X magnification; nuclei were counterstained with DAPI; boxes indicate areas shown at higher magnification below to the corresponding panel; images were acquired by confocal microscope with 10×, 20× and 40× magnification with scale bars 200 μm (10×), 100 μm (20×), 50 μm (40x). **d** mRNA relative expression of senescence markers *CDKN1A*, *GLB1*, *IL6* and *IL8* in BOs exposed to SIPS agents alone (H_2_O_2_, blue bar or DOXO, plum bar) or combined with CCS1477 (CCS + H_2_O_2_, light blue bar; CCS + DOXO, pink bar), expressed as fold change calculated on their respective controls (untreated CTRL, dark gray bar and CCS, gray striped bar); dots express biological triplicates (*n* = 9) and values are expressed as means ± SEM. Representative western blot showing total protein abundance of senescent markers p21 and p53 normalized on TUJ1 (**e**) and replicates analysis (**f**) on BOs (BO D65) untreated (CTRL, dark gray bar) or exposed to H_2_O_2_ (blue bar), DOXO (plum bar), CCS (gray striped bar) or SIPS agents in combination with CCS (CCS + H_2_O_2_, light blue bar; CCS + DOXO, pink bar); dots represent *n* = 4 replicates and values are expressed as means ± SEM. Statistical analysis was performed using two-tailed Student *t*-test (*p < 0.05; **p < 0.01; ***p < 0.001).

Given the results obtained with iNeurons, we decided to leverage the 3D brain organoid model to confirm both the senescence induction by SIPS and the rescue by inhibiting p300 (Fig. 4 and Table 2, see Methods).

We differentiated brain organoids (BOs) starting from hiPSC following the adapted protocol to day 65 (Fig. 4a, b). To assess the expression of different neural markers and thus, the presence of neurons at distinct stages of differentiation, we characterized the brain organoids at day 65 by immunofluorescence (Fig. 4c). Specifically, NESTIN, a marker of neural precursors, is primarily expressed in brain organoid rosettes (Fig. 4c’), while the TUJ1 signal identifies immature neurons disseminated throughout the whole organoid (Fig. 4c’, c”). In addition, mature and cortical neurons represented by MAP2^+^ and CTIP^+^ cells tend to be located alongside rosettes and in the external part of the organoid (Fig. 4c”).

After establishing this 3D model, we exposed BOs to SIPS agents to induce senescence, alone or in combination with the p300 inhibitor, to rescue the senescent phenotype (Table 2, see Methods). We assessed the relative expression of mRNA for senescence markers such as *CDKN1A*, *GLB1*, *IL6*, and *IL8* by RT-qPCR (Fig. 4d), checking also the expression of p300 targets (Supplementary Fig. 6b). As previously observed for LCLs and iNeurons, both H_2_O_2_ and DOXO exposure led to an increased level of senescence markers in BOs, compared to their untreated condition. There were significantly higher levels of *CDKN1A*, *IL6* (for both transcripts *p* < 0.05 in H_2_O_2_ and *p* < 0.01 in DOXO treatments), and *IL8* (*p* < 0.01 for both SIPS treatments) as shown (Fig. 4d). Remarkably, transcript levels of senescence markers decreased when SIPS treatments were combined with the p300 inhibitor (CCS + H_2_O_2_ or CCS + DOXO) when compared to exposure to SIPS agents only in BOs (*p* < 0.001 and *p* < 0.01 in H_2_O_2_-treatments for *CDKN1A* and *IL8* respectively, and *p* < 0.05 in DOXO-treatments for *IL8*) (Fig. 4d).

In addition, we investigated senescence marker expression by western blotting (Fig. 4e and Supplementary Fig. 7b) as we performed for all our models previously described in this study. When BOs were treated with SIPS agents, we observed an increase of p21 (*p* < 0.05 for the samples treated with H_2_O_2_ and with CCS + DOXO compared to their respective controls CTRL and CCS) and p53 (*p* < 0.05 for CCS + DOXO compared to CCS-treated BOs). Interestingly, the p21 and p53 increase appeared less marked upon CCS and SIPS agents cotreatment (CCS + H_2_O_2_ or CCS + DOXO) compared to SIPS agents alone (H_2_O_2_ or DOXO, respectively), significant for H_2_O_2_-treatments (*p* < 0.01 for p21 and *p* < 0.05 for p53 expression) (Fig. 4f).

Together, these experiments showed that a senescent-like phenotype can be rescued through p300 inhibition by CCS1477 in a 3D in vitro model of the CNS, i.e., brain organoids.

### p300 inhibition by CCS1477 counteracts transcriptional senescent cascade in Brain Organoids (BOs)

**Fig. 5:**
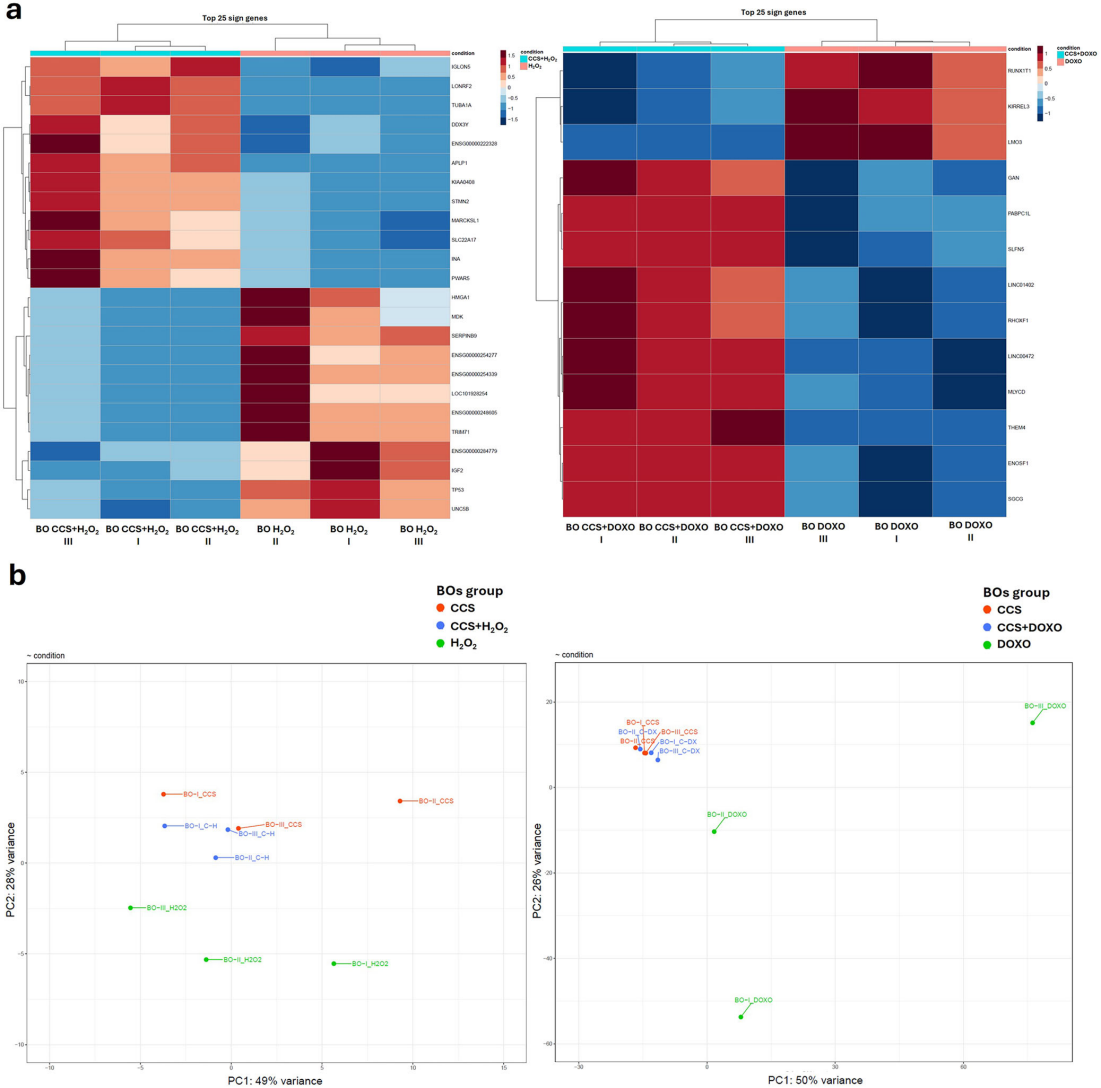
p300 inhibition by CCS1477 rescues the transcriptional senescent cascade induced by SIPS agents in Brain Organoids (BOs). **a** Heatmaps of whole transcriptome RNA-seq expression data showing top 25 DEGs in biological triplicates of BOs (marked with I, II, III) each treated with SIPS agents alone (H_2_O_2_ or DOXO) or combined with CCS1477 (CCS + H_2_O_2_ or CCS + DOXO); comparison of H_2_O_2_-treated and DOXO-treated BOs are shown on the left and on the right panels respectively; DEGs were selected based on nominal *p* < 0.05 and absolute foldchange > 1.5. **b** PCA plots of transcriptomic analysis on biological triplicates of BOs (marked with I, II, III) each treated with CCS1477 (CCS), SIPS agents alone (H_2_O_2_ or DOXO) or combined with CCS1477 (CCS + H_2_O_2_ or CCS + DOXO); comparison between CCS with H_2_O_2_-treated and DOXO-treated BOs are shown on the left and on the right plots respectively; on axes is shown the percentage of each principal component (PC1 on *x*-axis, PC2 on *y*-axis) that explains the population variance.

Considering the role of p300 in transcriptional regulation, we studied the whole BOs transcriptome upon both SIPS induction alone and p300 inhibitor cotreatment to verify the rescue of the induced senescent-like phenotype at the transcriptional level (Fig. 5). Firstly, we confirmed by gene set enrichment analysis (GSEA) that SIPS agents (H_2_O_2_ and DOXO) globally suppressed transcription of genes involved in proliferation and synaptic activity compared to control BOs (CTRL) (Supplementary Fig. 9). In addition, significant differences were observed in selected hallmark gene sets from GSEA among BOs exposed to SIPS and control BOs (Supplementary Fig. 10a), while as expected none was found among the two SIPS agents (Supplementary Fig. 10b).

Interestingly, significant differentially expressed genes (DEGs) were observed when we compared SIPS induction (H_2_O_2_ or DOXO) with their respective cotreatment with CCS (CCS + H_2_O_2_ or CCS + DOXO) (Fig. 5a and Supplementary Fig. 11a), with an enrichment of genes associated with proliferation and neuronal activity as revealed by GSEA for gene ontology (GO) (Supplementary Fig. 12). Intriguingly, principal component analysis (PCA) showed cotreated BOs samples (CCS + H_2_O_2_ or CCS + DOXO) clustered closely to CCS-treated BOs samples, whereas not to H_2_O_2_− or DOXO- treated BOs (Fig. 5b), confirmed also by UMAP analysis (Supplementary Fig. 11b).

These findings suggest that the transcriptional cascade characterizing the senescent-like phenotype can be counteracted by p300 inhibition in BOs, highlighting DEGs and dysregulated pathways involved in the process.

## Discussion

The pathogenesis of age-related disorders has been widely studied and ascribed to multiple molecular processes, from chronic inflammation to mitochondrial dysfunction^32,33^. In the last decades, cellular senescence emerged as a characteristic of aging, and it has been linked to the development and progression of neurodegenerative diseases^34^. In addition, targeting senescent cells to ameliorate pathological features has proven to be effective for some age-related conditions^35^. Current strategies foresee the use of senolytic and senomorphic agents to induce apoptosis of senescent cells and suppression of SASP expression, respectively. The recent identification of p300 as an epigenetic driver of cellular senescence and an active player in a class of neurodegenerative diseases, such as tauopathies, renders its in-depth elucidation a high priority to remedy a significant data gap^23,36^. To date, the p300/CBP bromodomain inhibitor CCS1477 is the only inhibitor currently in phase 1b/2a clinical trials for the treatment of hematological malignancies and advanced drug-resistant prostate cancer^37–39^.

Herein, we reported that p300 inhibition by CCS1477 could ameliorate senescent-like phenotypes in 2D and 3D in vitro models of SIPS. Specifically, we induced DNA damage and senescence by ROS (hydrogen peroxide, H_2_O_2_) or the genotoxic compound (doxorubicin, DOXO) as stressor agents in all our models and tested CCS1477 (CCS) action on cellular senescence in combination with these treatments, assessing senescent-like phenotype with multiple markers.

We initially confirmed the p300 role in SIPS in *EP300*^*+/−*^ and HD LCLs treated with SIPS agents. Interestingly, H_2_O_2_− or DOXO-treated cells with p300 haploinsufficiency due to germline mutations (*EP300*^*+/−*^ LCLs) resulted in their being less sensitive to DNA damage compared to HD LCLs, showing fewer γH2AX^+^/Cas3^−^ cells by flow cytometry and decreased expression of senescent markers (*CDKN1A*, *GLB1*, p21 and p53) by RT-qPCR and western blots compared to controls.

Next, we tested CCS1477 (CCS) in HD LCLs and IMR90 cells, an established in vitro model for senescence studies. We confirmed CCS action in HD LCLs, observing a significant reduction of H3K27 acetylation in CCS-treated cells compared to the ones untreated or treated with the vehicle. We also observed a relative decreased expression of p300 transcriptional targets (*MYC* and *POLD2*) in both CCS-treated HD LCLs and IMR90 compared to their respective controls. Remarkably, when HD LCLs were treated with CCS in combination with SIPS agents (CCS + H_2_O_2_ or CCS + DOXO), they expressed fewer γH2AX^+^/Cas3^-^ cells, a relative reduced expression of *CDKN1A*, *GLB1* and *IL6* transcripts, and decreased p21 and p53 total protein abundance, compared to the HD LCLs treated with only H_2_O_2_- or DOXO-treatments. These data suggest that p300 inhibition by CCS1477 protects HD LCLs from DNA damage induced by SIPS agents.

We were interested in understanding the impact of p300 inhibition in SIPS cells in a neuronal context; therefore, as a 2D model, we used iNeurons, which were treated with SIPS agents and with CCS in combination with H_2_O_2_ or DOXO. Intriguingly, there was a decreasing trend in SA-β-Galactosidase activation, as assessed by chromogenic staining, and in SASP expression at the transcriptional (*CDKN1A*, *LMNB1*, *IL6*, and *IL8*) and protein (p21 and p53) levels in iNeurons treated with CCS combined with SIPS agents compared to cells treated with stressor agents only. Thus, we demonstrated that p300 inhibition by CCS1477 can also rescue the senescent-like phenotype induced by SIPS agents in iNeurons.

We also explored SIPS and p300 inhibition in brain organoids (BOs) after characterizing the establishment of this 3D model system. We used D65 BOs that had not yet undergone the physiological senescence process in order to assess an induced premature senescent state^40^. Initially, we confirmed SIPS in BOs treated with stressor agents, as attested by the significant increase of mRNA (*CDKN1A*, *GLB1*, *IL6*, and *IL8*) and protein (p21 and p53) expression of senescent markers compared to controls. Notably, similar to what was seen in the iNeuron experimental model, the administration of the p300 inhibitor could clearly rescue the senescent-like phenotype induced in BOs, based upon a decreasing expression of senescent markers in the BOs undergoing CCS treatment combined with SIPS compounds compared to the BOs treated with stressor agents alone.

In addition, we investigated BOs transcriptome upon SIPS and CCS1477 (CCS) co-treatment. After SIPS effects confirmation at the transcriptional level, we evaluated the impact of CCS in combined treatments compared to single-SIPS agent exposure, identifying several significant DEGs. Interestingly, among the upregulated transcripts in cotreated BOs, we found many genes associated with neurodegenerative disorders (*IGLON5*, *GAN*, and *STMN2*)^41–43^, as well as downregulated senescence-associated genes already reported in other in vitro models (*HMGA1*, *IGF2*, *MDK*, *TP53, UNC5B*, *LMO3*)^44–47^. Strikingly, PCA results showed that the cotreatment with CCS clustered with CCS treatment alone and not with the samples exposed to SIPS agents only, suggesting that p300 inhibition can rescue the transcriptional cascade induced by SIPS.

Thus, in this work, we demonstrated in both 2D and 3D in vitro models that the inhibition of p300 using CCS1477 could represent a promising strategy for targeting senescent cells/senescence modulation and prevention. Our findings suggest that p300 may serve as a possible target in therapeutic approaches for treating age-related diseases. In conclusion, we have explored the effects of p300 inhibition in sophisticated neuronal models of SIPS, which could pave the way for its assessment also in in vivo disease models. We observed H3K27ac increase, and given the known effects on key senescence drivers (e.g., p21, p53, etc.)^48^ and the senescent phenotype in our models, we speculated a p300 modulation of SIPS phenotype. Clearly, the possibility to modulate SIPS by p300 inhibition is intriguing, however, further experiments are needed to unveil the precise mechanism of action. This approach could be explored for age-related disorders (e.g., neurodegenerative diseases); throughout the characterization of dysregulated gene environments associated with specific diseases, we can potentially repurpose existing approved medications or elaborate combined-treatment strategies^49,50^. The existence of a gene network convergence between cognition and neurodevelopment has been documented^51^, suggesting that investigating molecules reported to be dysfunctional in disorders affecting nervous system growth and maturation could identify new targets, representing collateral avenues, for therapeutic approaches in the neurodegenerative disorders field.

## Methods

### Experimental models

#### Lymphoblastoid cell lines (LCLs)

Lymphoblastoid cell lines (LCLs) derived from four patients carrying *EP300* mutations (*EP300*^*mut*^) and four healthy donors (HD) were obtained from the Telethon Network of Genetic Biobanks (Gaslini Genetic Bank, Genova, IT) (Table 1)^52–55^, mycoplasma tested and their use was approved by Ethical Committee of Università degli Studi di Milano (Comitato Etico number 99/20, 17 November 2020). Cells were cultured at 37 °C with 5% CO_2_ in RPMI 1640 medium (#ECM2001L, Euroclone) with L-glutamine (#ECB3004D, Euroclone), 20% fetal bovine serum (FBS, RM10432, Microtech), and penicillin/streptomycin (P/S, 100X, #ECB3001, Euroclone). Both *EP300*^*mut*^ and HD LCLs were treated with the stressor agents hydrogen peroxide (H_2_O_2_, #95321, Sigma-Aldrich) or doxorubicin (DOXO, #S1208, Selleckchem) and their respective vehicles (H_2_O and DMSO, respectively). In addition, HD LCLs were treated with CCS1477 (CCS, #CT-CCS1477, ChemieTek), a known p300 bromodomain inhibitor, a combination of CCS with one of the two agents mentioned above (CCS + H_2_O_2_ or CCS + DOXO), and their respective vehicles as described in Table 2.

**Table 1:** *EP300*^*mut*^ LCLs used for in vitro treatments

Gene	*EP300*^*mut*^ LCLs	cDNA change	Protein change	Mutation type	Reference
***EP300***	RSTS 25	c.41_51delinsT	p.(K14Ifs*31)	Frameshift	Negri et al. ^52^
	RSTS 39	c.4640dupA	p.(N1547Kfs*3)	Frameshift	Negri et al. ^53^
	RSTS 54	c.669dupT	p.(Q223Sfs*19)	Frameshift	Negri et al. ^52^
	RT010-15	c.4763T > C	p.(M1588T)	Missense (HAT)	Di Fede et al. ^54^

HAT histone acetyltransferase domain.

**Table 2:** Conditions of in vitro treatments used on experimental models

2D and 3D models treatments	Hydrogen peroxide (H_2_O_2_)	Doxorubicin (DOXO)	CCS1477 (CCS)
Vehicle	H_2_O	DMSO	DMSO
Concentration	100–**300**–500 µM	50–**100** nM–50 µM	100–300–**500** nM
Time for 2D models	**2**–6–24 h	**24**–48–72 h	**24**–48–72 h
Time for 3D model	**6**–12–24 h	24–48–**72** h	24–48–**72** h

#### IMR90

IMR90 cells (ATCC CCL-186), which are primary fetal lung fibroblasts, were grown in culture medium made of EMEM (#AL047S, HiMedia Laboratories), 10% FBS, and P/S as previously described. These cells were tested for Mycoplasma and treated as described for HD LCLs.

#### iNeurons

Healthy donor hiPSC line JBWT2.3 was kindly provided by Dr. Ian Krantz, mycoplasma tested and maintained as feeder-free cells in E8 Flex medium (#A2858501, Thermo Fisher Scientific) and 1% penicillin/streptomycin on Geltrex-coated dishes (#A1413201, Thermo Fisher Scientific). For generating a stable cell line capable of rapid and efficient neuronal differentiation, we utilized a PiggyBac construct carrying an inducible Neurogenin-2 (NGN2) overexpression cassette^56^. To generate stable hiPSC-NGN2 cell lines, 2.25 µg of PiggyBac construct (ePB-Bsd-TT-Ngn2-EGFP-Puro, obtained from the Diego Pasini laboratory) and 250 ng of the plasmid containing the transposase were transfected using Lipofectamine 3000 (#STEM00008, Thermo Fisher Scientific), according to the manufacturer’s protocol. After four days of 5 µg/ml blasticidin selection (#ant-bl-05, InvivoGen), 2000 cells were seeded in a 15-cm dish, and clones were picked after another week. Clone screening was carried out by inducing neuronal differentiation with 2 µg/ml of doxycycline (#D9891, Sigma-Aldrich) and selecting the clones expressing GFP during differentiation.

For neuronal differentiation, stable generated hiPSC-NGN2 were seeded in Induction medium (Advanced DMEM/F12, #12634010, Thermo Fisher Scientific) supplemented with N2 (100X, #17502048, Thermo Fisher Scientific), Non-Essential Amino (NEAA, 100X, #ECB3054D, Euroclone), L-glutamine (100X), P/S (100X), and 2 µg/ml of doxycycline on Geltrex-coated dishes with 10 µM of Y-27632 (ROCK inhibitor, ROCKi, #S1049, Selleckchem) (day 0). On day 1 the culture medium was changed to an Induction medium without ROCKi, while on day 2 the cells were placed on an Induction medium with 1 µg/ml of puromycin (#ant-pr-1, InvivoGen). On day 3, pre-neurons were accutase-dissociated (#A1110501, Thermo Fisher Scientific) and seeded on 10 µg/ml of Laminin (#23017015, Thermo Fisher Scientific) and 50 µg/ml of poly-D-lysine-coated dishes (#A3890401, Thermo Fisher Scientific) in Cortical medium composed by Neurobasal plus (#A3582901, Thermo Fisher Scientific) supplemented with B27 (50X, #A3582801, Thermo Fisher Scientific), 10 ng/ml of BDNF (#450-02, Thermo Fisher Scientific), 10 ng/ml of NT-3 (#450-03, Thermo Fisher Scientific), P/S (100X) and 2 µg/ml of doxycycline, with 10 µM of ROCKi. On day 4, the medium was changed to Cortical medium without ROCKi, and the following weeks 50% of Cortical medium was changed twice a week until D16 or D23. iNeurons were treated as reported for HD LCLs. Biological replicates in experiments were obtained from different line batches.

#### Brain organoids (BOs)

Forebrain Organoids (BOs) were generated from hiPSC line JBWT2.3 using a modified version of already published protocols^57,58^. Briefly, 10,000 cells/well were seeded in a U-bottom 96 low-attachment multi-well in E8 flex medium with ROCKi (10 µM) and centrifuged for 5 min at 270 × *g* (day 0). On day 2, the medium was changed with E8 flex medium containing SB-431542 (10 µM) (#1614, Tocris Bioscience) and Dorsomorphin (5 µM) (#P5499, Sigma-Aldrich) and completely changed every two days until day 6. From day 6 to day 22, BOs were cultured in Neurobasal medium (#A3582901, Thermo Fisher Scientific) with Glutamax (100X, #35050061, Thermo Fisher Scientific), P/S (100X), B27-Vitamin A (50X, #12587010, Thermo Fisher Scientific) supplemented with EGF (20 ng/mL) (#AF-100-15, Thermo Fisher Scientific) and FGF-2 (20 ng/mL) (AF-100-18B, Thermo Fisher Scientific). At day 12 the BOs were placed in a 24 low-attachment multi-well and half medium changed every 2/3 days. From day 22 to day 45, BOs were grown in Neurobasal medium with Glutamax (100X), P/S (100X), B27-Vitamin A (50X) supplemented with BDNF (20 ng/mL), NT-3 (20 ng/mL), ascorbic acid (AA, 200 μM) (#49752, Sigma-Aldrich), Dibutyryl cAMP sodium salt (D-cAMP, 50 μM) (#28745-M, Sigma-Aldrich), Docosahexaenoic Acid (DHA, 10 μM) (#1715505, Sigma-Aldrich). From day 45 to day 65, the BOs were maintained in Neurobasal medium supplemented with B27 (50X, #17504044, Thermo Fisher Scientific).

At day 65, BOs were collected or treated as reported in Table 2. Biological replicates in experiments were obtained from different line batches.

### Flow cytometry

LCL fixation and permeabilization for flow cytometry were performed using CytoFix/Perm Kit (#554714, BD Biosciences) according to manufacturer’s instructions. Briefly, cellular pellets were washed two times with PBS and incubated with Fixation solution on ice for 20 min. PBS was added to samples, and they were centrifuged to remove the Fixation solution. After another wash in PBS, cellular pellets were incubated with Permeabilization solution at room temperature (RT) for 20 minutes. The solution was removed by centrifugation, pellets were resuspended in Permeabilization solution, and labeled with the antibody anti-phospho γH2A.X FITC-conjugated (250 ng for each sample) (#560445, BD Biosciences) and anti-activated Cleaved Cas3 APC-conjugated (125 ng for each sample) (#560626, BD Biosciences). In parallel tubes with normal mouse IgG isotype control FITC- and APC-conjugated, and one tube with no label were prepared. Samples were incubated for 45 min at RT in the dark at which point they were washed with Wash Buffer. For each experiment, both biological and technical duplicates were performed. Tubes were read at BD FACSVerse^TM^ flow cytometer, data acquisition and analysis were performed using the BD FACSuite v1.0.6 software.

### RNA extraction and gene expression analysis

RNA extraction from LCLs, IMR90, iNeurons pellets, and BOs (pool of minimum two BOs/tube) was obtained by adding 1 mL of NucleoZOL (#FC1740404, Macherey-Nagel) to each sample for nucleoprotein complex dissociation. After chloroform/isopropanol purification and precipitation, RNA pellets were washed two times with 75% ethanol. Once dried, the pellets were resuspended in nuclease-free water, and the RNA was quantified by Nanodrop. All-In-One 5X RT MasterMix (#G592, Applied Biological Materials) was used for reverse transcription (RT), according to the manufacturer’s protocol, retrotranscribing 1 µg of RNA per sample into cDNA. The RT reaction products were diluted with H_2_O, and quantitative real-time PCR (RT-qPCR) was carried out using *TB Green* Premix Ex Taq (Tli RNase H Plus) (#RR420W, Takara Bio) and the *CFX Opus 96 Real-Time PCR System (#12011319, Bio-Rad)*. Experiments were assayed in biological and technical triplicates, and the expression levels of each gene of interest were normalized by three housekeeping genes (*GAPDH*, *RPLP0*, *RPL13A*)^59^ and quantified relative to the expression of untreated samples (CTRL) or treated with p300 inhibitor (CCS) using a comparative ΔΔCt quantification method. The primers used for RT-qPCR are listed in Table 3.

**Table 3:** List of primers used in this work for RT-qPCR

Gene	Sequence 5’-3’ Forward	Sequence 5’-3’ Reverse
*GAPDH*	AGCCACATCGCTCAGACAC	GCCCAATACGACCAAATCC
*RPLP0*	TCTACAACCCTGAAGTGCTTGAT	CAATCTGCAGACAGACACTGG
*RPL13A*	CCTGGAGGAGAAGAGGAAAGAGA	TTGAGGACCTCTGTGTATTTGTCAA
*CDKN1A*	GACACCACTGGAGGGTGACT	CAGGTCCACATGGTCTTCCT
*LMNB1*	GAACCAGAACTCGTGGGGCA	ATGCTCTTGGGGTTCCCTGC
*GLB1*	AATCAAGACCGAAGCAGTGGC	GGGTGAGTTGGCCCCATTCC
*IL6*	CCGGGAACGAAAGAGAAGCT	GCGCTTGTGGAGAAGGAGTT
*IL8*	CTTTCCACCCCAAATTTATCAAAG	CAGACAGAGCTCTCTTCCATCAGA
*MYC*	TCTGAGGAGGAACAAGAAGA	CAGCAGAAGGTGATCCAGAC
*POLD2*	ATAAGGCCAAATACCTCACC	GAGCGTGTAATTGGTGGGA
*STMN2*	AGCTGTCCATGCTGTCACTG	GGTGGCTTCAAGATCAGCTC
*LMO3*	ACTGTGCTTACTGAACGGCCTC	CCGGTCCTTGATCTTTCGGTTG

### Protein extraction and western blot

LCLs and iNeurons pellets and BOs (pool of minimum four BOs/tube) were washed in PBS, the dry samples were resuspended in cold S300 buffer (50 mM HEPES pH 7.6, 300 mM NaCl, 0.1% NP40, 2 mM MgCl_2_, 10% glycerol) supplemented with a protease inhibitor cocktail (#P8340, Sigma-Aldrich) and benzonase nuclease (#SC-202391, Santa Cruz Biotechnology), and left on ice for 1 h. Samples were centrifuged at maximum speed (13,000 rpm) for 10 min at 4 °C, and the supernatant was quantified with Bradford assay following the manufacturer’s instructions. Protein samples were denatured in Laemmli sample buffer 4x (LSB, #1610747, Bio-Rad) supplemented with β-mercaptoethanol (#1610710, Bio-Rad) and boiled for 10 min at 100 °C prior to gel loading.

Protein samples were separated by SDS-PAGE (Running buffer 1x diluted from 10x made of 3% Tris HCl, 14,4% Glycine and 1% SDS) and transferred to nitrocellulose membranes (#10600003, Cytiva) in Transfer buffer 1x (20% methanol and 10% Transfer buffer 10x, composed of 3% Tris HCl and 14,4% Glycine). Membranes were washed with TRIS-buffered saline (TBS 1x diluted from 10x made of 3% Tris HCl, 8,7% NaCl and 0,2% KCl) supplemented with 0.1% Tween (TBS-T), blocked for 1 h at RT with 5% milk in TBS-T and then with primary antibody diluted in blocking solution or TBS-T for 1 h at RT (rabbit anti-GAPDH, 1:2000, Cell Signaling #5174; anti-Tubulin β 3, TUJ1, 1:2000, BioLegend #801201) or overnight at 4 °C (rabbit anti-p21, 1:1000, Cell Signaling #2947S; mouse anti-p53, 1:1000, Invitrogen MA512557). Membranes were washed in TBS-T and incubated with goat anti-rabbit or anti-mouse IgG horseradish peroxidase (HRP)-conjugated antibodies (#1706515 and #1706516, Bio-Rad) diluted in blocking solution or TBS-T for 1 h at RT. After membrane washes, chemiluminescence signals were detected through ECL incubation (#1705061, Bio-Rad and #RPN2232, Cytiva) and captured by a Chemidoc Imaging System. Data obtained from western blots were analyzed by Image Lab Software and expressed as the ratio between values from proteins of interest and from housekeeping markers (GAPDH or TUJ1). Experiments were performed in a minimum of biological and technical duplicates.

### AlphaLISA assay

An amount of 1 × 10^4^ cells/well was resuspended in 60 µl of RPMI for AlphaLISA assay (PerkinElmer)^54^. AlphaLISA Cellular Detection Kit for Acetylated-Histone H3 Lysine 27 (H3K27ac) (AL720, PerkinElmer), normalized on unmodified Histone H3 Lysine 4 (H3K4) (AL719, PerkinElmer), was used for the acetylation assessment. Briefly, after incubation of biological triplicates with Cell-Histone Lysis buffer and Cell-Histone Extraction buffer for 15 and 10 min, respectively, technical triplicates were incubated with Acceptor mix for 1 h at RT, followed by the addition of Donor mix and incubation overnight at RT. Signal was detected using a PerkinElmer Ensight™ plate reader.

### Senescence-associated β-galactosidase (SA-β-Gal) staining

Treated IMR90 cells and iNeurons were fixed and stained for β-galactosidase marker using Senescence β-galactosidase staining kit (#9860S, Cell Signaling Technologies) according to the producer’s protocol. Briefly, the medium was removed, and the cells were washed with PBS. They were incubated with Fixative solution for 15 min at RT, washed twice with PBS, and finally incubated overnight at 37 °C in a dry incubator with Staining solution containing 1 mg of X-gal substrate for each sample. The following day, three to five images per sample of a randomly selected field from at least biological and technical duplicates of the stained cells were acquired by brightfield microscopy at 4× and 10× magnification. Three different blinded operators utilizing ImageJ software analyzed the microscopic images.

### Immunofluorescence labeling

Cells and BOs were fixed with 4% paraformaldehyde (PFA) for 15’ at RT or 1 h and 30’ at 4 °C, respectively. Then, BOs were cryopreserved with 30% sucrose overnight; the day after they were embedded in OCT and stored at –80 °C until use. Sections of 20 μm were obtained by cryostat.

For immunofluorescence labeling, hiPSCs, pre-Neurons, iNeurons, and BO sections were permeabilized for 10’ with PBT (PBS with 0.1% for pre-Neurons and iNeurons or 0,5% Triton 100X for iPSCs and CO sections) and incubated 30’ (hiPSCs, pre-Neurons, and iNeurons) or 1 h (BOs) at RT with blocking solution (PBT 0.1% or 0,5% and 10% natural donkey serum). The primary antibodies rabbit anti-NANOG (1:200, #4903, Cell Signaling), mouse anti-OCT4 (1:200, Cell Signaling #75463), mouse anti-SOX2 (1:50, Invitrogen MA1-014), rabbit anti-NESTIN (1:100, Abcam ab176571), mouse anti-Tubulin β 3 (TUJ1 clone, 1:1000), rabbit anti-CTIP (1:100, Cell Signaling BK12120S), goat anti-MAP2 (1:100, Abcam ab302487), rabbit anti-p21 (1:400, Cell Signaling 2947S) were used.

Secondary antibodies Alexa Fluor 488- cy3- or 647- conjugated donkey anti-goat, anti-mouse, and anti-rabbit Fab fragments (1:200; Jackson Immunoresearch) were used. DAPI (1:1000) was used for nuclei counterstaining.

### Microscope acquisitions

Brightfield images of hiPSCs, pre-neurons, iNeurons, and brain organoids were acquired with 4× and 10× magnification. Confocal images were acquired with a Nikon A1R/AX laser scanning confocal microscope equipped with a Nikon A1/AX plus camera and the following objectives (Nikon): Plan Fluor 10X DIC L N1 (NA 0.3), Plan Fluor 20X DIC N2 (NA 0.5), 20X Plan Apo λD OFN25 DIC N2 (NA 0.80), 40X Apo LWD WI λS DIC N2 (NA 1.15), 60X Plan Apo λD OFN25 DIC N2 (NA 1.42). DAPI, Alexa 488, Cy3, and Alexa 647 were excited at 405, 488, 561 nm, and 640 nm and observed at 425–475, 500–550, 570–620, and 663–738 nm, respectively.

### Transcriptome analysis

RNA extracted from biological triplicates of BOs was also used for whole-transcriptome RNA-sequencing (RNA-Seq) analysis, using the Universal plus Total RNA-Seq kit with targeted transcript depletion with AnyDeplete for globin genes (#9156, Tecan). The yields of final libraries were assessed by Qubit 4.0 fluorimeter, and their sizes were measured by Agilent Bioanalyzer. The libraries were analyzed by paired-end sequencing on NextSeq2000 Illumina platform, 2×100. FASTQ files are available in the ArrayExpress database (www.ebi.ac.uk/arrayexpress) under accession numbers E-MTAB-14464.

Raw FASTQ sequences were quality-tested with FastQC^60^ and aligned against the GRCh38/hg38 reference human genome with the splice-aware aligner STAR v2.7.10b^61^. Sorted, indexed BAM alignment files were used for quantification with featureCounts (v2.0.0)^62^, considering only uniquely mapped reads. GRCh38 Ensembl Release 108 annotation was used as reference.

The Bioconductor package DESeq2 v1.30^63^ was used to perform differential gene expression analysis in addition to custom shell and R scripts. Significant gene sets were selected based on nominal *P* value < 0.05 and absolute FoldChange > 1.5.

### Statistical analyses

Data were analyzed using Prism software (GraphPad Software v.10.2.2) and expressed as mean ± Standard Error of the Mean (SEM) or ±Standard Deviation (SD). Statistical analyses on flow cytometry and SA-β-Gal staining counts, RT-qPCR, and western blot data were performed using two-tailed Student *t*-test, considering significance for *p* value < 0.05 (*p < 0.05; **p < 0.01; ***p < 0.001).

## Supplementary information

Supplementary information

## Data Availability

FASTQ files are openly available in the ArrayExpress database (www.ebi.ac.uk/arrayexpress) under accession numbers E-MTAB-14464.
