# Supplementary material for: p300 inhibition delays premature cellular senescence
Source: NPJ Aging. 2025 Jul 10;11(1):62. doi: 10.1038/s41514-025-00251-y (PMC12246142; doi:10.1038/s41514-025-00251-y)
Supplement: Supplementary file 1 — Supplementary information [file 41514_2025_251_MOESM1_ESM.pdf]

SUPPLEMENTARY INFORMATION

p300 inhibition delays premature cellular senescence

Elisabetta Di Fede[1,2], Esi Taci[1,2], Silvia Castiglioni[1], Stefano Rebellato[3,4], Silvia Ancona[1], Paolo Grazioli[1], Chiara Parodi[1], Elisa A. Colombo[1], Clara Bernardelli[1], Elena Lesma[1], Ian D. Krantz[5,6], Stefania Corti[7,8], Alberto Priori[1,2], Grazia Fazio[3,4], Cristina Gervasini[1,2,$], Valentina Massa[1,2,$,*], Antonella Lettieri[1,$]

1 Department of Health Sciences, Università degli Studi di Milano, 20142, Milan, Italy
2 "Aldo Ravelli" Center for Neurotechnology and Experimental Brain Therapeutics, Università degli Studi di Milano, 20142, Milan, Italy
3 Tettamanti Center, Fondazione IRCCS, San Gerardo dei Tintori, 20900, Monza, Italy
4 School of Medicine and Surgery, University of Milano-Bicocca, 20900, Monza, Italy
5 Division of Medical Genetics, Cohen Children's Medical Center, Northwell Health, 11021, New York, USA
6 Department of Pediatrics, Zucker School of Medicine, Hofstra University, 11549, New York, USA
7 Dino Ferrari Centre, Department of Pathophysiology and Transplantation (DEPT), Neuroscience Section, University of Milan, 20122, Milan, Italy
8 IRCCS Foundation Ca' Granda Ospedale Maggiore Policlinico, 20122, Milan, Italy

$ These authors contributed equally to this work

* **Corresponding author**

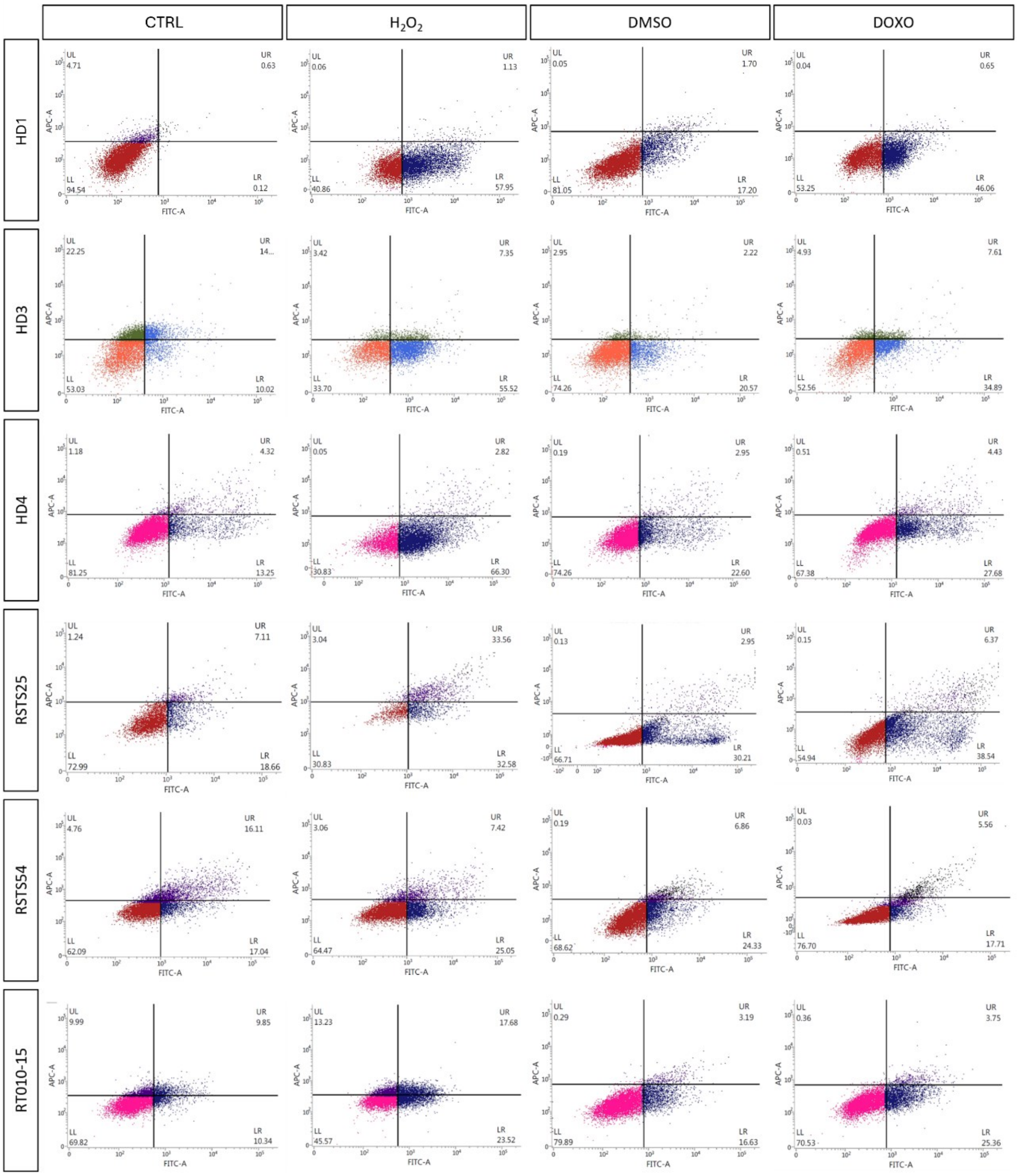

Supplementary Fig. 1 Representative flow cytometry panel for DNA damage induced by SIPS agents in *EP300^{mut}* and HD LCLs. Panel showing γH2AX (FITC-A) and cleaved Caspase 3 (APC-A) evaluation of LCLs derived from three individuals (RSTS25, RSTS54 and RT010-15) of the cohort with germline pathogenic variants in *EP300* (*EP300^{mut}*) and from three individuals (HD1, HD3 and HD4) of the control cohort (HD) both untreated (CTRL), treated with hydrogen peroxide (H_2O_2), doxorubicin (DOXO) or its vehicle (DMSO); for each cell line there are four quadrants showing percentage of cells negative for both APC and FITC signals (lower left, quadrant LL), positive for APC and negative for FITC signal (upper left quadrant, UL), positive for both (upper right quadrant, UR) or negative for APC and positive for FITC signal (lower right quadrant, LR).

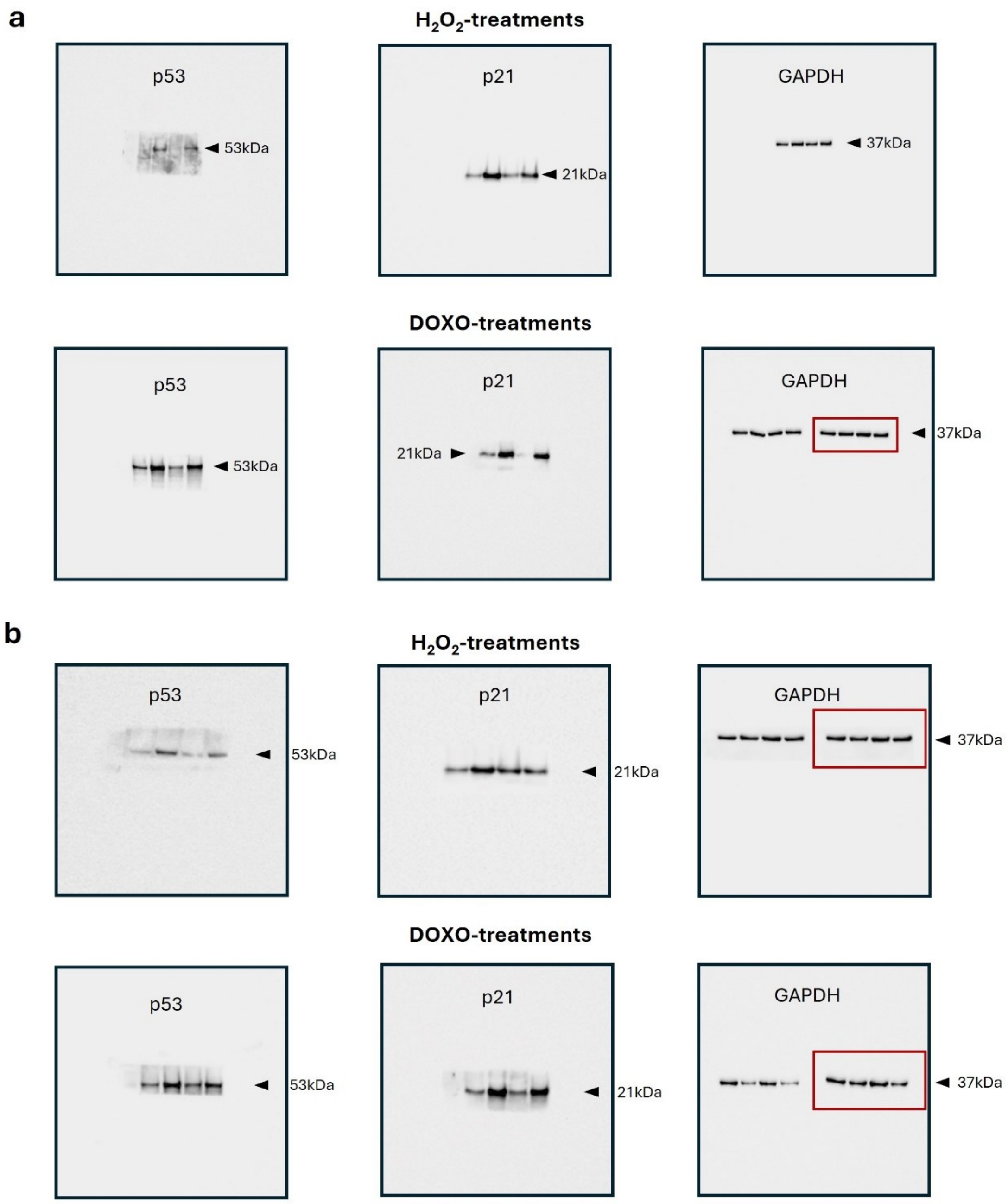

Supplementary Fig. 2 Unprocessed blot images related to Figures 1 and 2. Western blot images relative to Fig. 1d (**a**) and Fig. 2f (**b**).

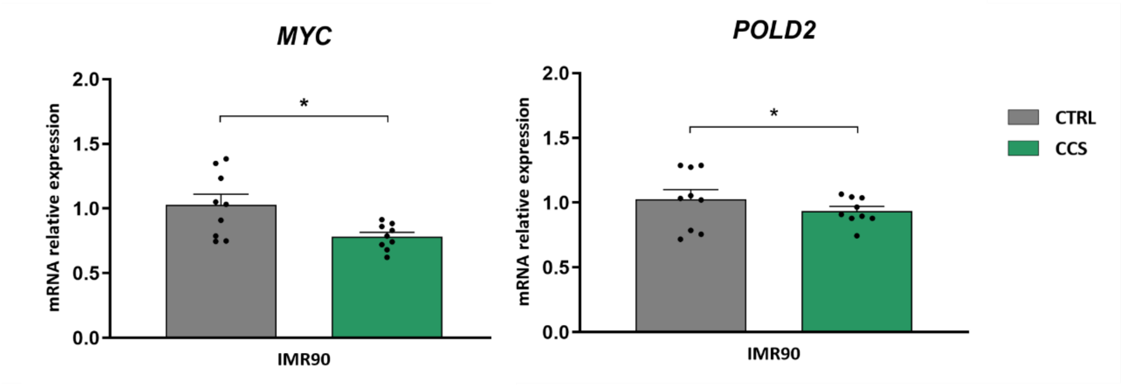

Supplementary Fig. 3 Evaluation of p300 inhibition by CCS1477 in IMR90 cells. mRNA relative expression of known p300 transcriptional targets *MYC* and *POLD2* in IMR90 cells, untreated (CTRL, grey bar) and treated with CCS1477 (CCS, green bar); dots express individual values of n=3 biological triplicates and values are expressed as mean ± SEM. Statistical analysis was performed using two-tailed *Student* t-test (* $p < 0.05$; ** $p < 0.01$; *** $p < 0.001$).

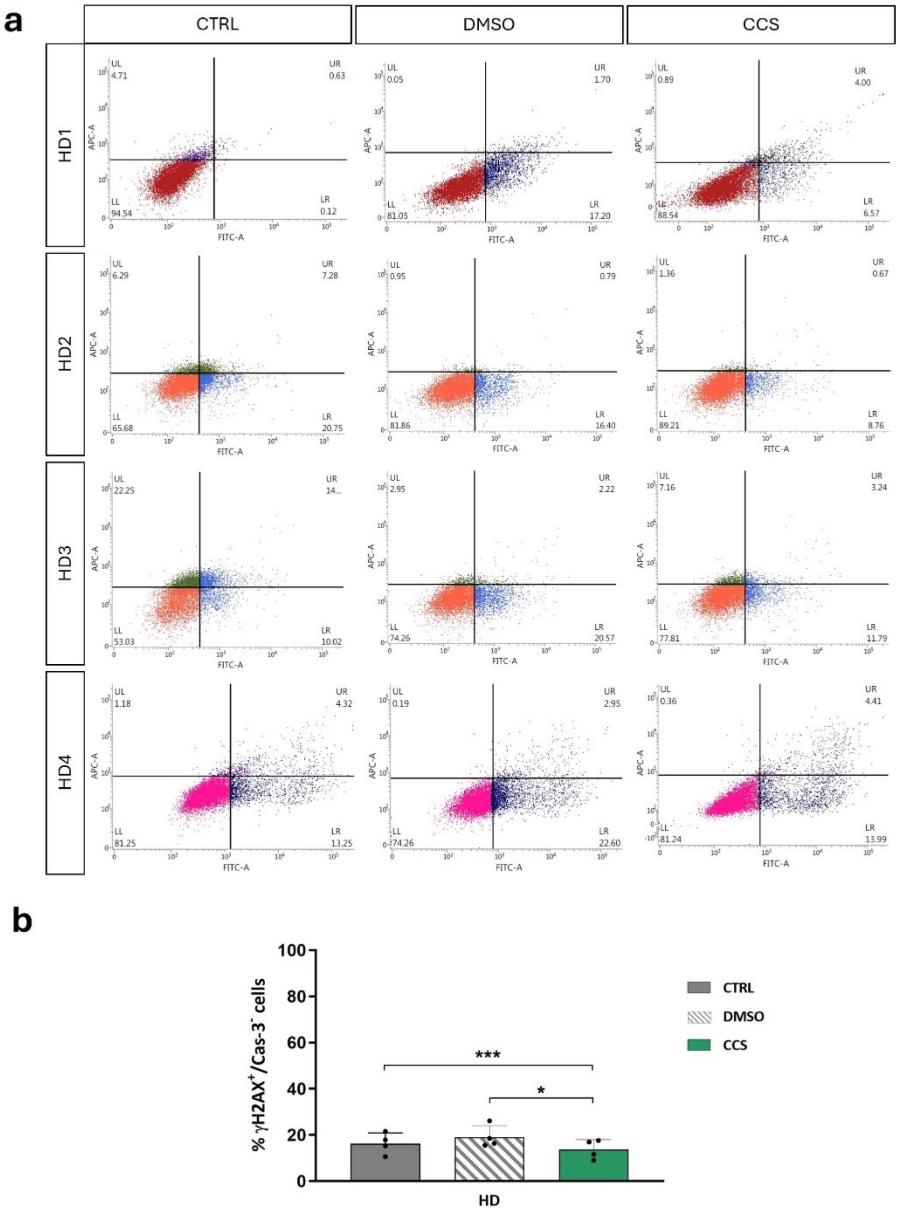

Supplementary Fig. 4 Cell viability in HD LCLs upon CCS1477 treatment. **a,** Representative flow cytometry panel of γH2AX (FITC-A) and cleaved Caspase 3 (APC-A) evaluation of four LCLs derived from healthy donors (HD1, HD2, HD3 and HD4) untreated (CTRL), treated with p300 inhibitor (CCS) or its vehicle (DMSO); for each cell line there are four quadrants showing percentage of cells negative for both APC and FITC signals (lower left, quadrant LL), positive for APC and negative for FITC signal (upper left quadrant, UL), positive for both (upper right quadrant, UR) or negative for APC and positive for FITC signal (lower right quadrant, LR). **b,** Flow cytometry analysis showing percentage of cells positive to γH2AX not positive to cleaved Caspase 3 (% $\gamma H2AX^{+}/Cas\text{-}3^{-}$ cells, on Y axis) on HD lines, not treated (CTRL, grey), treated with the vehicle (DMSO, grey stripes) or p300 inhibitor (CCS, green); each dot represents the mean of triplicates of *n*=4 cell lines and values are expressed as mean ± SD. Statistical analysis was performed using two-tailed *Student* t-test (* $p < 0.05$; ** $p < 0.01$; *** $p < 0.001$).

a

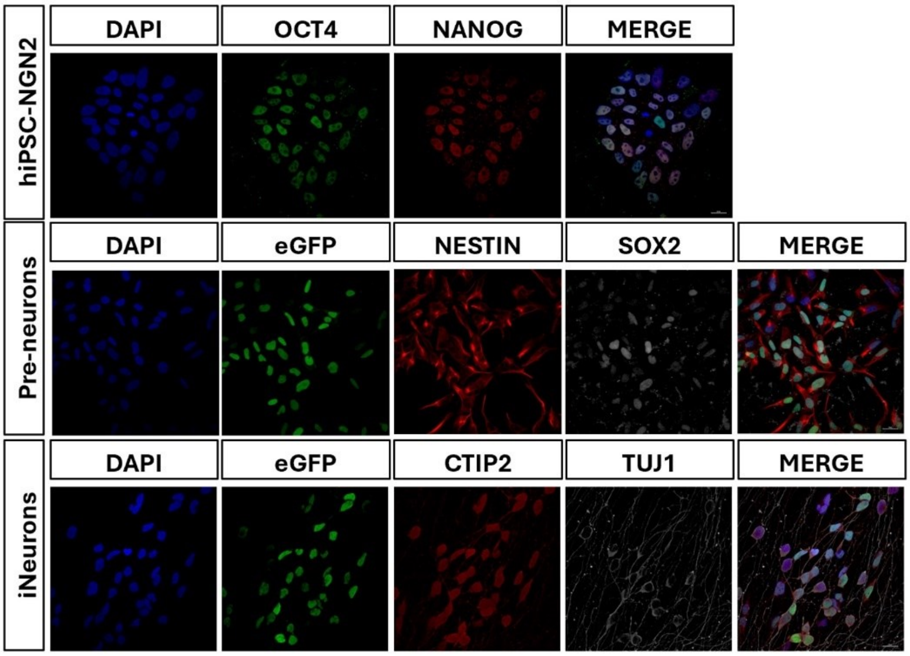

b

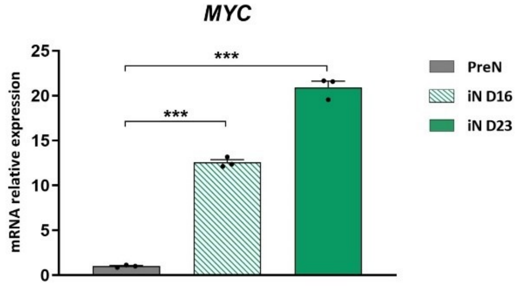

Supplementary Fig. 5 iNeurons characterization for differentiation and aging stages. **a,** Characterization of three main differentiation stages of iNeurons (hiPSC-NGN2, Pre-neurons and iNeurons) by immunofluorescence experiments, images were acquired using a confocal microscope with 60x magnification; confocal images show hiPSCs marked with OCT4 (green signal) and NANOG (red signal), Pre-neurons with NESTIN (red signal) and SOX2 (white signal), iNeurons with CTIP (red signal) and TUJ1 (white signal), all nuclei with DAPI (blue signal), while endogenous GFP due to inducible differentiation (eGFP, green signal) was detected in both Pre-Neurons and iNeurons. **b,** mRNA relative expression of known p300 transcriptional targets *MYC* in Pre-neurons (PreN), iNeurons at D16 (iN D16, light green bar) and at D23 (iN D23, dark green bar) of differentiation; dots show n=3 replicates and values are expressed as mean ± SEM. Statistical analysis was performed using two-tailed *Student* t-test (* $p < 0.05$; ** $p < 0.01$; *** $p < 0.001$).

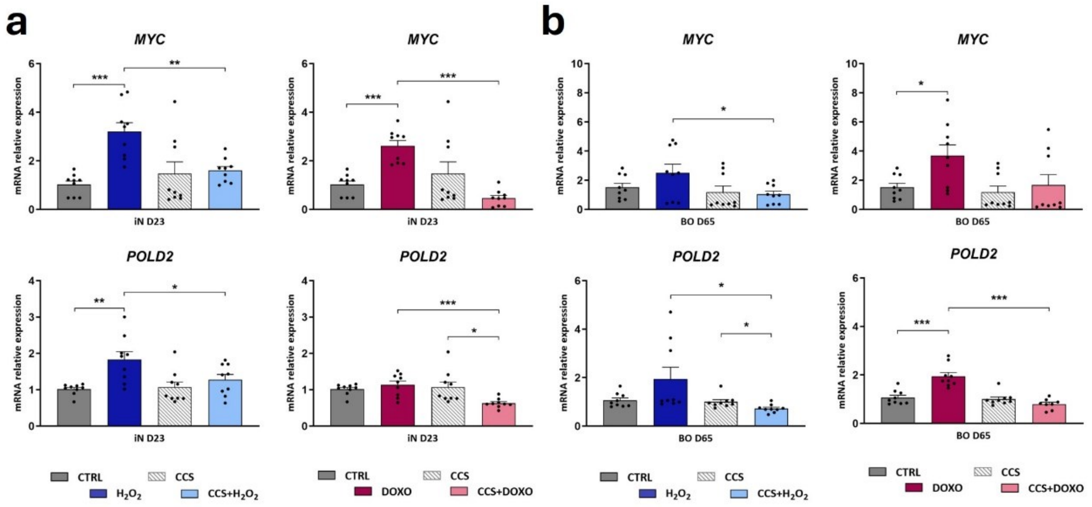

Supplementary Fig. 6 p300 inhibition by CCS1477 downregulates p300 transcriptional targets in iNeurons (iN) and Brain Organoids (BOs) treated for modeling SIPS. **a-b,** mRNA relative expression of p300 transcriptional targets *MYC* and *POLD2* in iNeurons (**a**) and BOs (**b**) exposed to SIPS agents alone (H_2O_2, blue bar or DOXO, plum bar) or combined with CCS1477 (CCS+H_2O_2, light blue bar; CCS+DOXO, pink bar), expressed as fold change calculated on their respective controls (untreated CTRL, dark grey bar and CCS, grey striped bar); dots express biological triplicates (n=9) and values are expressed as means ± SEM. Statistical analysis was performed using a two-tailed *Student* t-test (* $p < 0.05$; ** $p < 0.01$; *** $p < 0.001$).

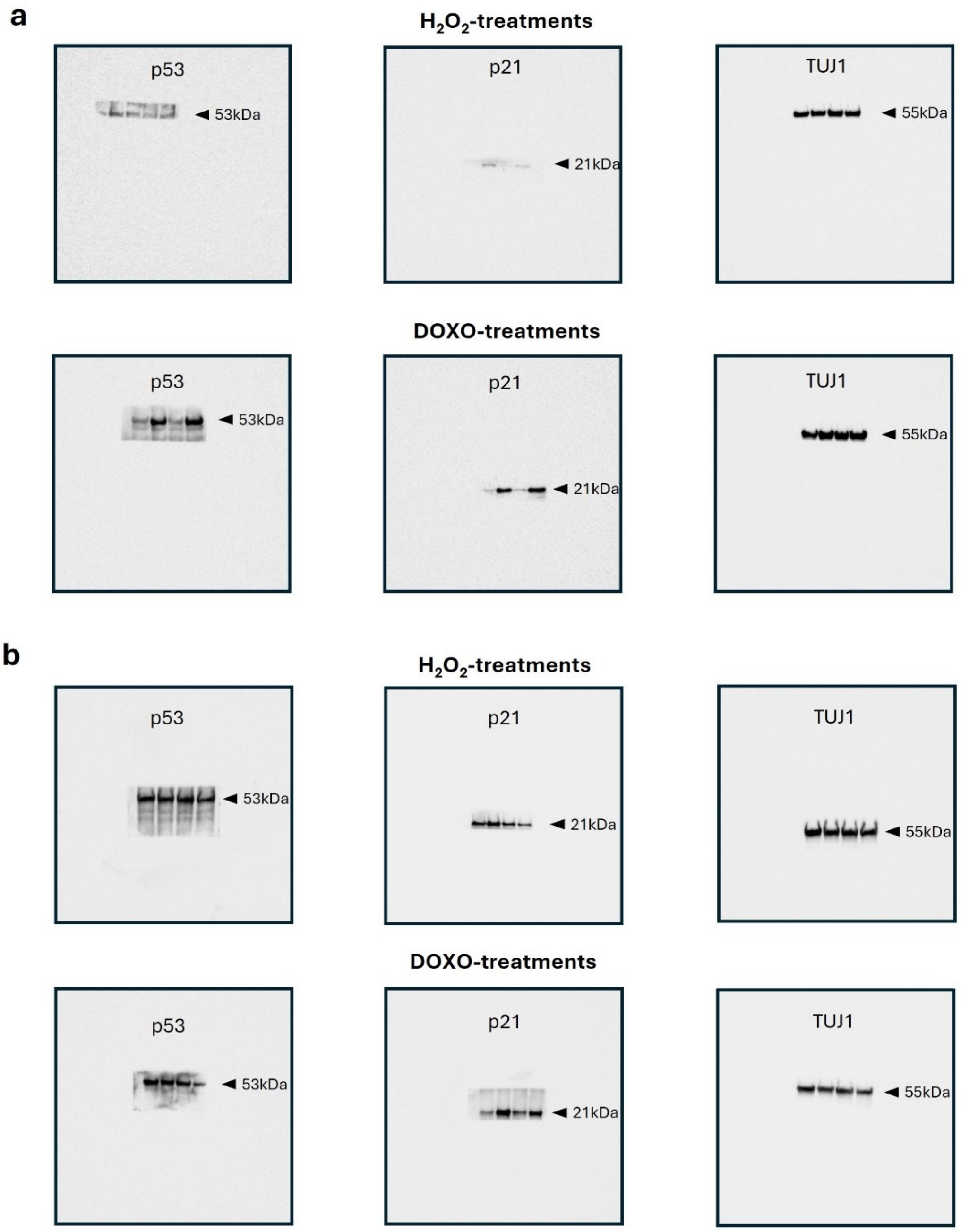

Supplementary Fig. 7 Unprocessed blot images related to Figures 3 and 4. Western blot images relative to Fig. 3e (**a**) and Fig. 4e (**b**).

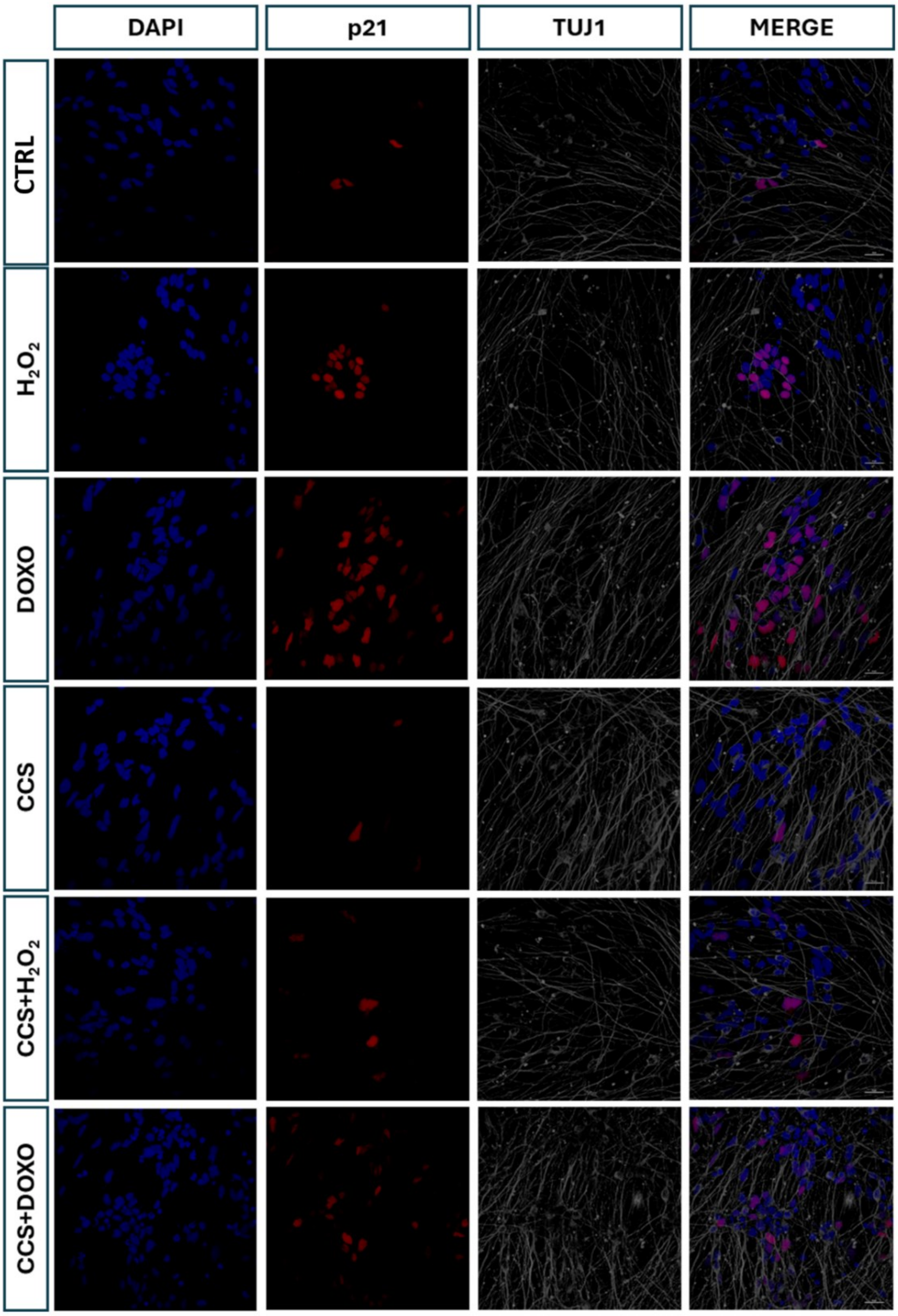

Supplementary Fig. 8 p21 expression in iNeurons senescence-like phenotype induced by SIPS agents and rescued by CCS1477. Confocal images of immunofluorescence experiments at 60x magnification showing iNeurons untreated (CTRL), treated with SIPS agents (H_2O_2 or DOXO), p300 inhibitor (CCS) or CCS combined with SIPS agents (CCS+ H_2O_2 or CCS+DOXO), all marked with p21 (red signal), TUJ1 (white signal) and DAPI (blue signal).

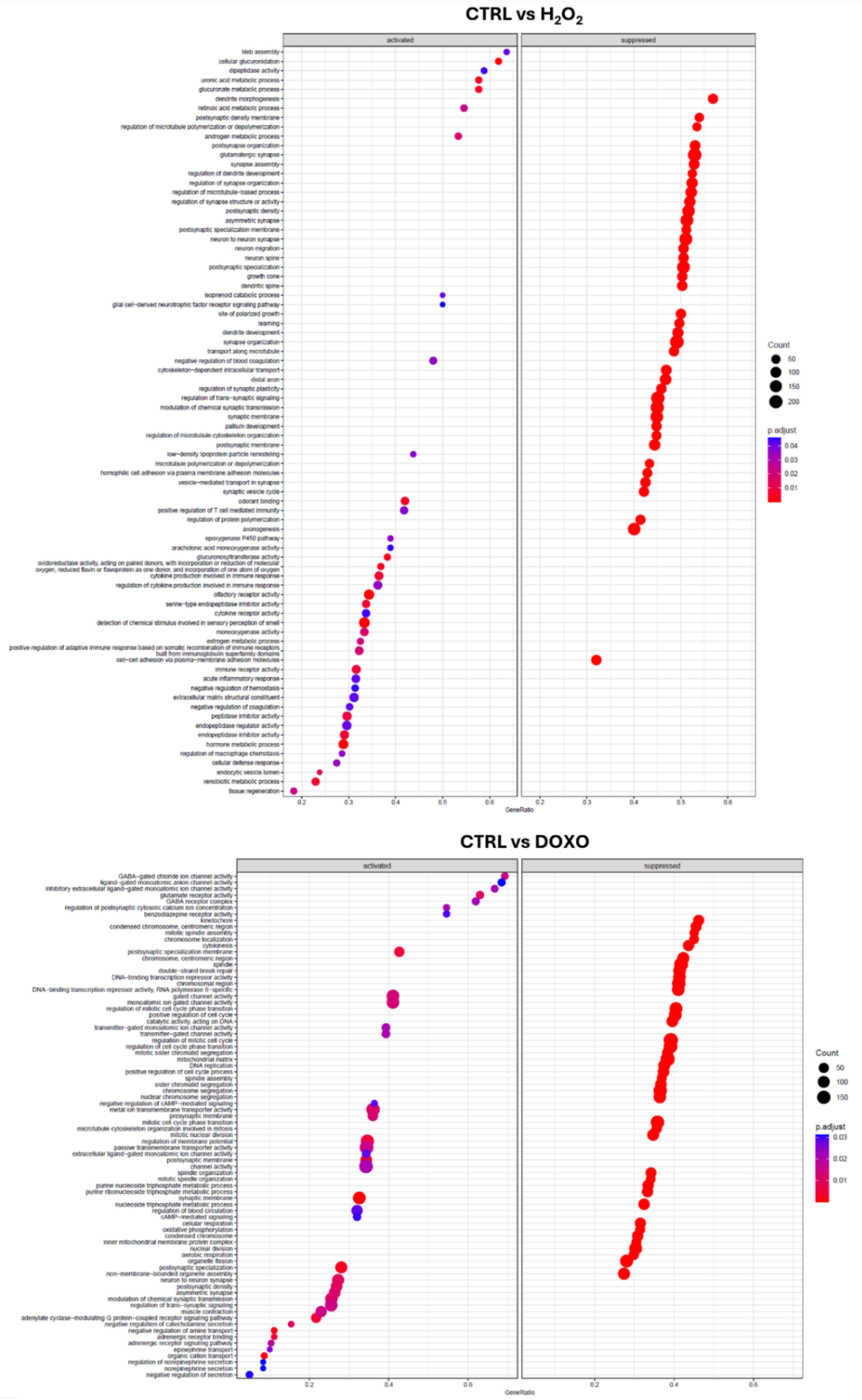

Supplementary Fig. 9 Gene Set Enrichment Analysis (GSEA) terms for Gene Ontology (GO) in BOs. GSEA in BOs with senescence-like phenotype induced by SIPS agents (H_2O_2, upper panel, and DOXO, bottom panel), both compared to control BOs (CTRL).

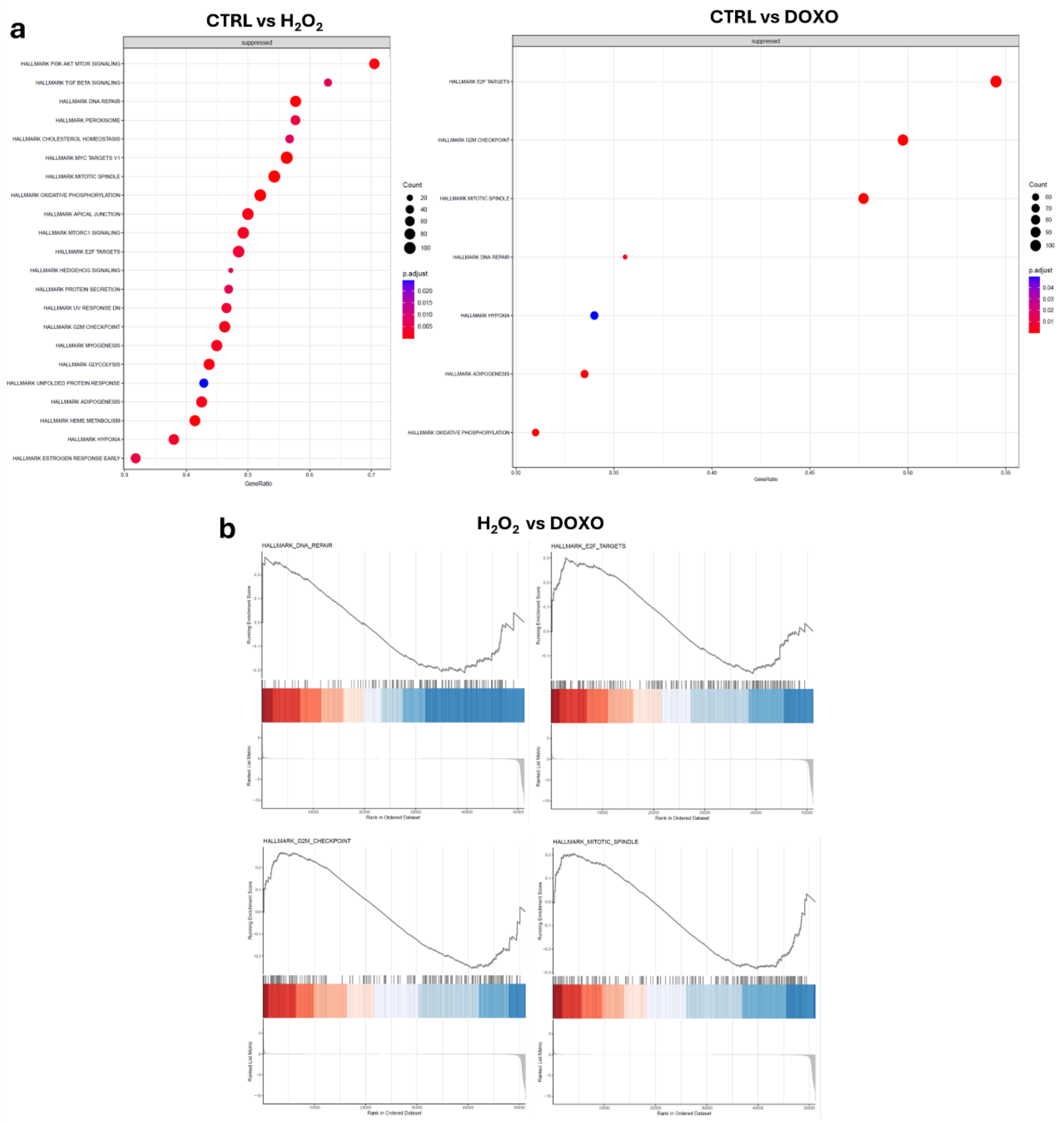

Supplementary Fig. 10 Hallmarks defined by Gene Set Enrichment Analysis (GSEA) in SIPS induction in BOs. **a,** Suppressed enrichment hallmarks terms in senescent-like phenotype induced by SIPS agents (H_2O_2, left panel, and DOXO, right panel) in BOs, both compared to control BOs (CTRL). **b,** GSEA plots for selected hallmark gene sets from Human MSigDB Collections reveal no significant differences among the two SIPS agents used for BOs SIPS induction (H_2O_2 vs DOXO).

a

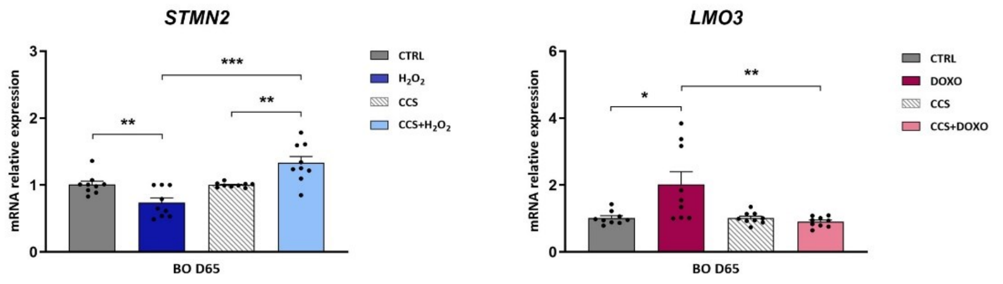

b

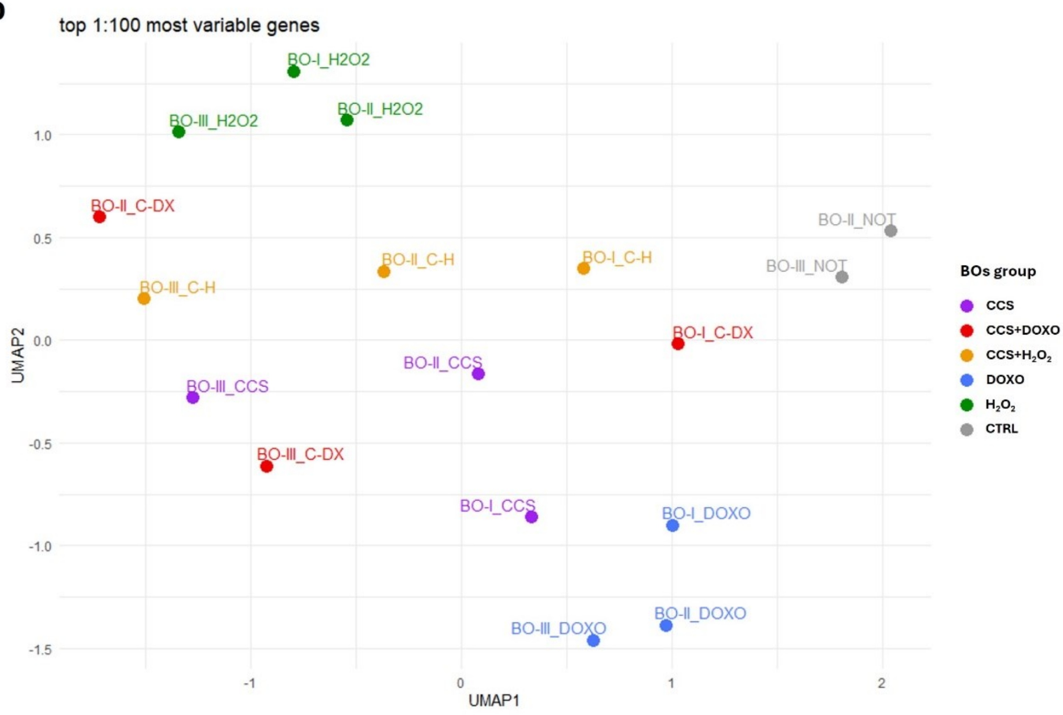

Supplementary Fig. 11 Validation of DEGs from RNA-seq results in BOs and representation of transcriptomic analysis performed for BOs. **a,** mRNA relative expression of the two DEGs *STMN2* and *LMO3* in BOs exposed to SIPS agents alone (H_2O_2, blue bar or DOXO, plum bar) or combined with CCS1477 (CCS+H_2O_2, light blue bar; CCS+DOXO, pink bar), expressed as fold change calculated on their respective controls (untreated CTRL, dark grey bar and CCS, grey striped bar); dots express biological triplicates (*n*=9) and values are expressed as means ± SEM. Statistical analysis was performed using a two-tailed *Student* t-test (* $p < 0.05$; ** $p < 0.01$; *** $p < 0.001$). **b,** UMAP plot of transcriptomic analysis on biological triplicates of BOs (marked with I, II, III) each untreated (CTRL), treated with CCS1477 (CCS), SIPS agents alone (H_2O_2 or DOXO) or combined with CCS1477 (CCS+H_2O_2 or CCS+DOXO); UMAP was performed using the *uwot* R package to compare all treatment groups, based on the top 100 most variable genes; the analysis was conducted with the following parameters: n_neighbors = 4, min_dist = 0.3, metric = "euclidean", and seed = 1.

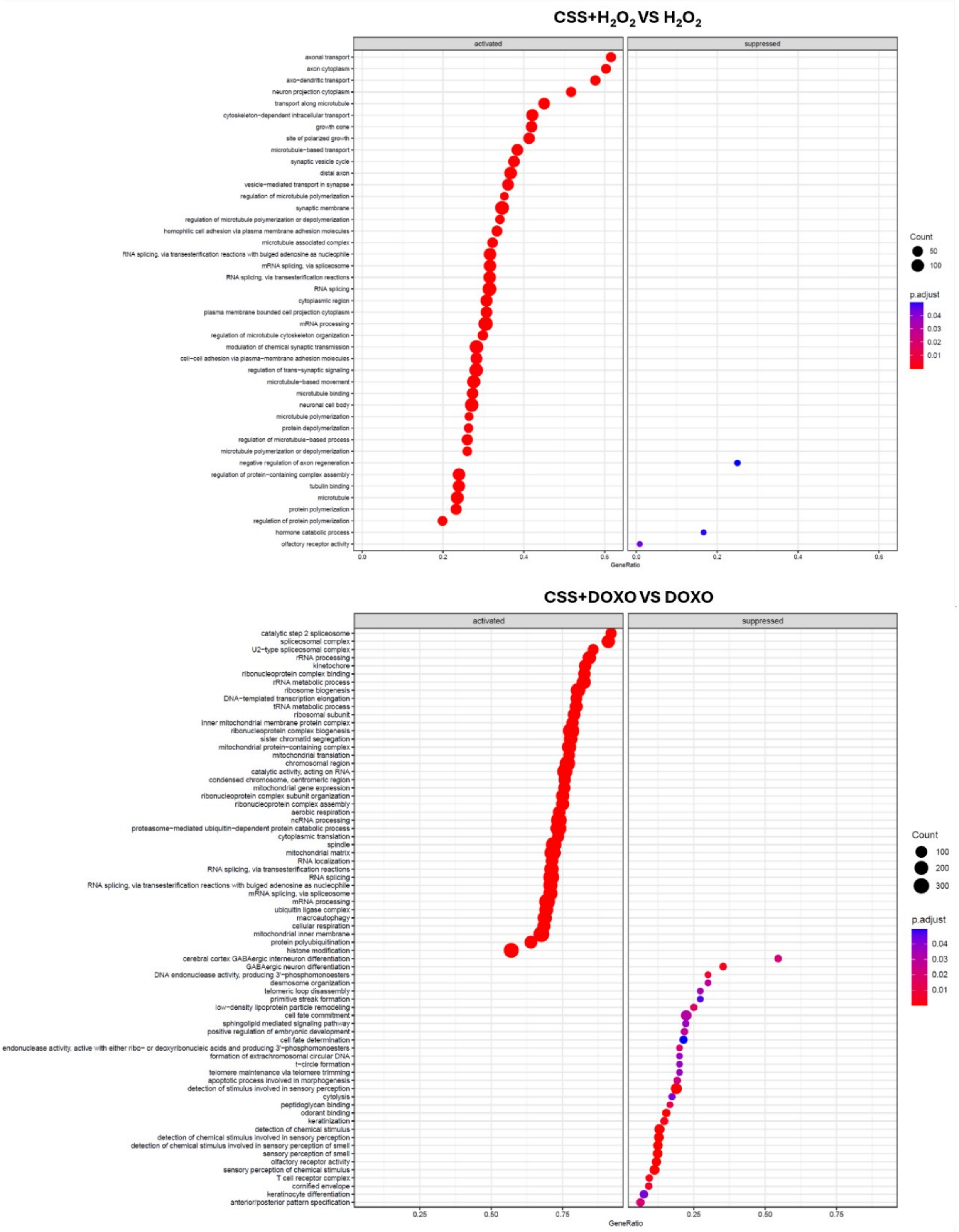

Supplementary Fig. 12 Gene Set Enrichment Analysis (GSEA) terms for Gene Ontology (GO) in BOs. GSEA in BOs with senescence-like phenotype induced by SIPS agents both compared to BOs cotreated with CCS (respectively CCS+H_2O_2 vs. H_2O_2, upper panel, and CCS+DOXO vs. DOXO, bottom panel).
